# SLX4 Prevents GEN1-Dependent DSBs During DNA Replication Arrest Under Pathological Conditions in Human Cells

**DOI:** 10.1038/srep44464

**Published:** 2017-03-14

**Authors:** Eva Malacaria, Annapaola Franchitto, Pietro Pichierri

**Affiliations:** 1 Section of Experimental and Computational Carcinogenesis, Department of Environment and Primary Prevention, Istituto Superiore di Sanità – Viale Regina Elena 299, 00161 Rome Italy; 2 Section of Molecular Epidemiology, Department of Environment and Primary Prevention, Istituto Superiore di Sanità – Viale Regina Elena 299, 00161 Rome Italy

## Abstract

SLX4 is a versatile protein serving as docking for multiple structure-specific endonucleases during DNA repair, however, little is known about its function at demised replication forks. Using RNAi or FA-P cells complemented with SLX4 mutants that abrogate interaction with MUS81 or SLX1, we show that SLX4 cooperates with MUS81 to introduce DSBs after replication stress but also counteracts pathological targeting of demised forks by GEN1. Such unexpected function of SLX4 is unrelated to interaction with endonucleases, but concerns the physical presence of the protein. Strikingly, ectopic expression of the Holliday junction-binding protein RuvA inhibits DSBs in SLX4-deficient cells by preventing GEN1 chromatin-association, and rescues proliferation and genome integrity upon replication stress. Altogether, our results indicate that SLX4 is crucial to prevent accidental processing of Holliday junction-like intermediates at demised forks also suggesting that spontaneous genome instability in FA-P cells may derive, at least partially, from unscheduled action of GEN1 in S-phase.

Multiple proteins can be engaged to rescue DNA synthesis at perturbed replication forks. Most of these proteins act to stabilize the replisome and promote the restart of replication avoiding the introduction of potentially-lethal DNA damage, such as DSBs^1^,^2^. However, if the fail-safe restart of the perturbed replication forks is not possible, as occurs in checkpoint-deficient cells or under other pathological conditions such as oncogene activation, more error-prone alternative mechanisms are triggered to promote cell survival^3^,^4^. Recently, it has been shown that MUS81, a structure-specific endonuclease (SSE) normally resolving recombination intermediates^5^,^6^, is required to process structures formed at perturbed forks under pathological replication^7^,^8^,^9^,^10^. This MUS81-dependent processing would support proliferation on pathological replication stress, however, it introduces genome instability^8^. During the resolution of recombination intermediates, the activity of MUS81 is stimulated or directed by the SLX4 protein, which acts as a scaffolding factor^12^,^13^,^14^. This function of SLX4 is conserved in yeast and humans, and may also be required to produce through the action of its partner SLX1 a nicked Holliday’s junction (HJ), which is the one of the preferred MUS81 substrates^6^,^15^. Whether the presence of SLX4/SLX1 activity is required to support MUS81-dependent cleavage also at demised replication forks in mammalian cells is unclear. Indeed, SLX4-depletion only partially reduces DSBs that accumulate in wild-type cells after checkpoint inhibition, but increases cell death in MUS81-depleted cells^9^. Moreover, at least after checkpoint inactivation, MUS81 might process a RAD52-dependent D-loop rather than a nicked HJ^9^, so that the SLX4 contribution to MUS81 function could be less relevant.

During mitotic processing of recombination intermediates, another SSE, GEN1(Yen1), can substitute for MUS81 or SLX4^16^,^17^. Even though GEN1(Yen1) shows ability to target also replication intermediates *in vitro*, its activity appears strictly prevented in S-phase in both yeast and human cells through CDK-dependent phosphorylation and nuclear exclusion^6^. In humans, however, if GEN1 can take over replication fork processing in absence of MUS81 under pathological conditions, such as checkpoint deficiency or oncogene activation, is unclear.

Thus, we analysed the contribution of SLX4 and GEN1 to replication fork processing into DSBs under pathological replication stress induced by CHK1 inhibition or oncogene activation. Our data evidence that GEN1 can produce DSBs after pathological replication stress in absence of MUS81, contributing to cell survival, however, they also indicate that such DSBs are prevented by the presence of SLX4, even if no other SSE is available to target demised replication forks.

Interestingly, MUS81, SLX4 and GEN1 have been found mutated or inactivated in human cancers (http://cancer.sanger.ac.uk/cosmic), and their expression levels in several cancer samples seems to be regulated in opposite way (http://www.proteinatlas.org/cancer). Furthermore, mutations in SLX4 have been associated to Fanconi anemia^18^,^19^. Hence, our findings contribute to shed light into pathological processing of perturbed replication forks in human cells. Moreover, they may be useful to our understanding of the correlation between replication stress and genomic instability in cancer as well as in genetic diseases.

## Results

### GEN1-dependent DSBs may form under pathological replication in absence of SLX4

**Figure 1:**
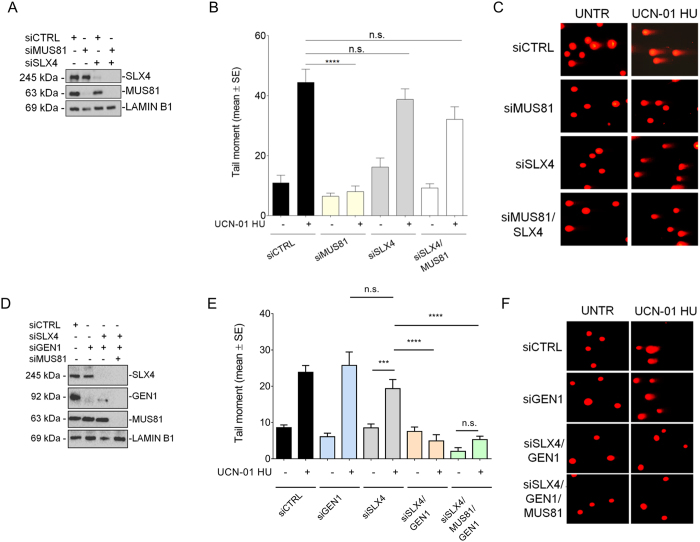
Loss of SLX4 function leads to MUS81-independent but GEN1-dependent DSBs formation after pathological replication stress. (**A**) Analysis of protein depletion in GM01604 cells after transfection with scrambled siRNA (siCTRL), siSLX4, siMUS81 alone or in combination. Immunoblotting was performed 48 h after transfection using the appropriate antibodies. LAMIN B1 was used as loading control. (**B**) Evaluation of DNA breakage by neutral Comet assay. Cells were treated with 600 nM UCN-01 and 2 mM HU for 6 h and then subjected to neutral Comet assay. Data are mean +/− standard errors (SE) from three independent experiments. (**C**) Representative images from selected samples are shown. (**D**) GM01604 cells were transfected with scrambled siRNAs (siCTRL) or siRNAs directed against GEN1 alone or in combination with siSLX4 and siMUS81. Cell lysates were subjected to immunoblotting with indicated antibodies. LAMIN B1 was used as loading control. (**E**) Formation of DSBs was analyzed with neutral Comet assay as in (**B**). Data are presented as mean +/− standard errors (SE) from three independent experiments. (**F**) Representative images of neutral comets from selected samples are shown. (ns = not significant p > 0.05; ***p < 0.001, ****p < 0.0001, Kruskal-Wallis test). Uncropped versions of gels are provided in Supplementary Material.

SLX4 and MUS81 act in a common pathway during resolution of recombination intermediates before mitosis^17^,^20^. Thus, we asked if depletion of SLX4 could affect formation of MUS81-dependent DSBs after pathological replication stress^8^,^9^. To this aim, we transfected hTERT-immortalised normal human primary fibroblasts with siRNAs targeting SLX4, MUS81 or both (Fig. 1A), and performed neutral Comet assay after treatment with UCN-01, a chemical CHK1 inhibitor, and HU a condition that induces replication fork demise and pathological replication stress^9^. As expected, treatment resulted in DSBs, which were suppressed by depletion of MUS81 (Fig. 1B,C). By contrast, and surprisingly, depletion of SLX4 did not significantly reduce the amount of DSBs after pathological replication stress, and co-depletion of SLX4 and MUS81 only slightly decreased the level of DNA breakage (Fig. 1B,C). However, after induction of DNA ICLs by mitomycin-C (MMC), depletion of SLX4, alone or in combination with that of MUS81, substantially reduced DSBs (Suppl. Fig. 1), in agreement with a cooperation between these two proteins in response to DNA ICLs^14^. Thus, our results demonstrating that MUS81-independent DSBs are induced upon replication fork demise but not after induction of ICLs in absence of SLX4, prompted us to verify whether other nucleases might substitute for MUS81 under pathological replication stress. During resolution of recombination intermediates, loss of MUS81 is overcome by GEN1^21^. Thus, we tested whether DSBs detected in SLX4-deficient cells in response to pathological replication stress were dependent on GEN1. We down-regulated GEN1 in cells depleted of SLX4 alone or in combination with MUS81 (Fig. 1D), and analysed formation of DSBs by neutral Comet assay after pathological replication stress (Fig. 1E,F). In wild-type cells, knockdown of GEN1 did not affect DNA breakage formed after treatment with UCN-01 and HU, however, it completely suppressed DSBs in cells depleted of SLX4 or MUS81+SLX4.

**Figure 2:**
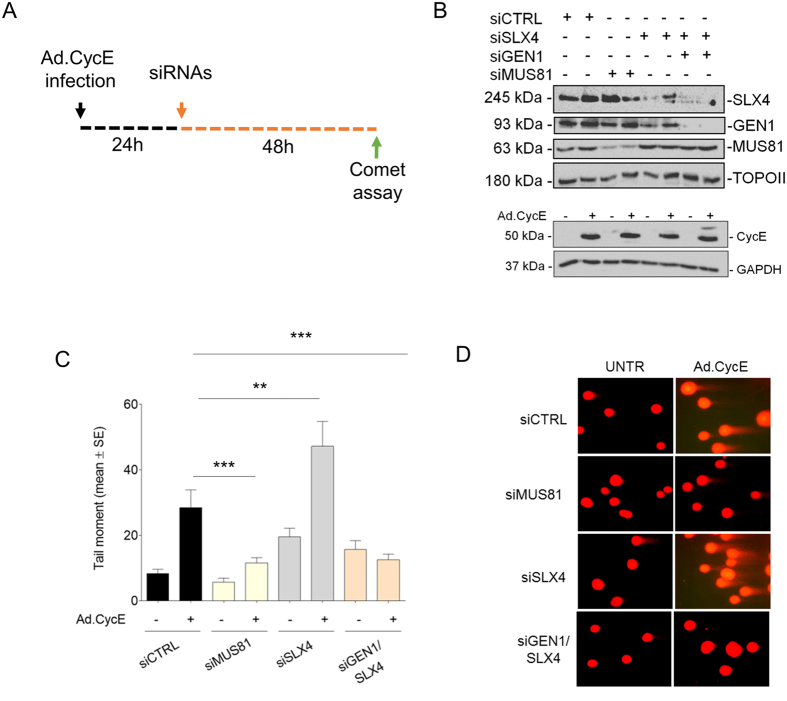
GEN1-dependent accumulation of DSBs in SLX4 depleted cells occurs after CycE overexpression. (**A**) Experimental scheme of CycE overexpression and siRNA transfection. (**B**) Cells were infected with Ad.Ctrl or Ad.CycE at 20 MOI, and CycE overexpression level, together with protein depletion, was assessed by immunoblotting using the indicated antibodies. GAPDH was used as loading control. (**C**) Evaluation of DSBs formation after CycE overexpression by neutral Comet assay. GM01604 cells were infected and transfected as in (**A**). Data are mean +/− standard errors (SE) from three independent experiments. (**D**) Representative neutral Comet assay images are shown in panels. (**p < 0.01, ***p < 0.001, Kruskal-Wallis test). Uncropped versions of gels are provided in Supplementary Material.

Given that formation of MUS81-dependent DSBs at replication forks is also triggered by oncogene-induced replication stress^8^,^10^, we investigated whether loss of SLX4 could result in taking over of demised replication forks by GEN1 also following oncogene activation. Thus, we overexpressed Cyclin E (CycE) at oncogenic level in hTERT-immortalised normal human primary fibroblasts^8^ and analysed formation of DSBs after downregulation of MUS81, SLX4, or SLX4+GEN1 by RNAi (Fig. 2A,B). Neutral Comet assay confirmed that oncogenic levels of CycE result in MUS81-dependent DNA breakage, and showed the persistence of DSBs in SLX4-depleted cells (Fig. 2C,D). Consistently with what observed in cells treated with UCN-01 and HU, co-depletion of SLX4 and GEN1 resulted in the suppression of DSBs caused by CycE overexpression (Fig. 2C,D).

GEN1 is assumed to be activated in mitosis to process late-recombination intermediates^22^. Thus, we verified whether loss of SLX4 could result in GEN1-dependent DSBs because of a premature mitotic entry. We depleted cells of SLX4 and analysed progression into mitosis by pS10-H3 immunostaining in the presence of nocodazole to accumulate mitotic cells (Suppl. Fig. 2A). Although treatment with UCN-01 alone resulted in a higher number of mitotic cells in both CTRL or SLX4-depleted cells, the combined UCN-01 and HU treatment determined a clear reduction of cell accumulating in mitosis as compared with the untreated cells and irrespective of the presence of SLX4. Moreover, co-immunofluorescence with anti-γ-H2AX and anti-BrdU antibodies confirmed that pan-nuclear γ-H2AX staining, a diagnostic sign of DSBs after replication stress, was detected almost completely in BrdU-positive cells (Suppl. Fig. 2B).

Altogether, these results indicate that GEN1 can generate DSBs in S-phase after replication stress, compensating for loss of MUS81, but only in absence of SLX4 and under conditions of pathological replication.

### Shielding of demised forks by SLX4 is sufficient to avert GEN1-dependent DSBs upon pathological replication arrest

**Figure 3:**
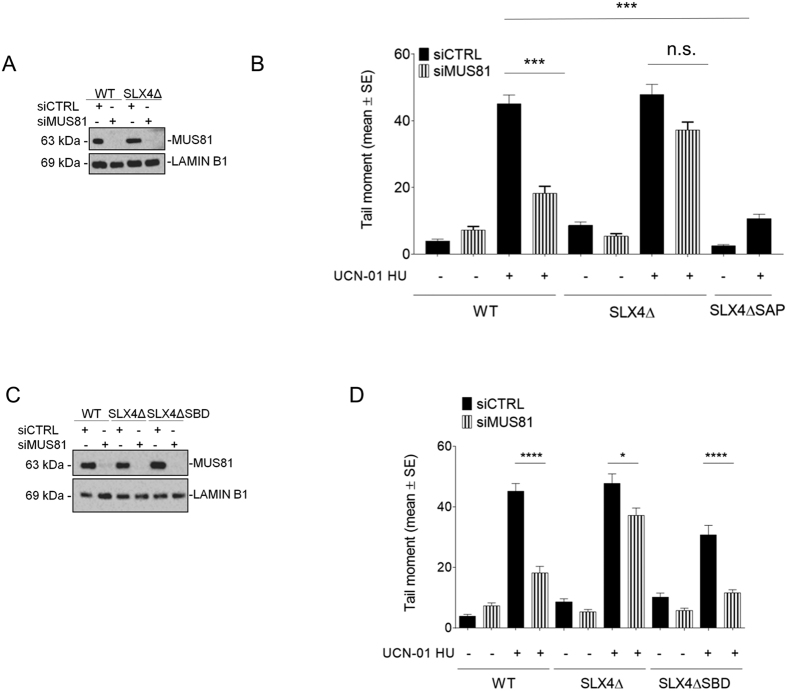
Abrogation of the SLX4-MUS81 or SLX4-SLX1 interaction does not stimulate GEN1-dependent DSBs formation. (**A**) Western immunoblotting showing MUS81 depletion in SLX4-deficient FA-P cells (SLX4Δ), complemented with wild-type SLX4 (WT). Lysates were prepared 48 h after transfection with indicated siRNA oligos. LAMIN B1 was used as a loading control. (**B**) SLX4-deficient FA-P cells (SLX4Δ), complemented with wild-type SLX4 (WT) or with the deletion mutant that abrogate MUS81 interaction (SLX4ΔSAP) were transfected with control siRNA (siCTRL) or siRNAs against MUS81 (siMUS81). The amount of DSBs was evaluated by neutral Comet assay after depletion of MUS81 and treatment with 600 nM UCN-01 and 2 mM HU for 6 h. Graph shows the mean tail moment +/− SE from three independent experiments. (ns = not significant p > 0.05; **p < 0.01, ***p < 0.001, Kruskal-Wallis test). (**C**) Western immunoblotting showing MUS81 depletion in SLX4-deficient FA-P cells (SLX4Δ), complemented with wild-type SLX4 (WT) or the SLX4 deletion mutant abrogating interaction with SLX1 (SLX4ΔSBD). Lysates were prepared 48 h after transfection with indicated siRNA oligos. LAMIN B1 was used as a loading control. (**D**) SLX4-deficient FA-P cells (SLX4Δ), complemented with wild-type SLX4 (WT) or with the deletion mutant that abrogates SLX1/SLX4 interaction (SLX4ΔSBD) were transfected with control siRNA (siCTRL) or siRNAs against MUS81 (siMUS81). The amount of DSBs was evaluated by neutral Comet assay after treatment with 600 nM UCN-01 and 2 mM HU for 6 h. Graph shows the mean tail moment +/− SE from three independent experiments. (ns = not significant p > 0.05; *p < 0.05; ****p < 0.0001, Kruskal-Wallis test). Uncropped versions of gels are provided in Supplementary Material.

That GEN1 takes over on MUS81 in absence of SLX4 might be simply due to impaired recruitment of MUS81 complex, which could unleash cleavage by GEN1. To exclude this possibility, we used RA3331/E6E7/hTERT cells, an isogenic pair of FA-P-derived transformed fibroblasts in which SLX4 is undetectable, expressing the wild-type form of SLX4 (WT) or an SLX4 deletion mutant that cannot associate with MUS81 (SLX4ΔSAP)^23^. These cells were depleted or not of MUS81 by RNAi and challenged with UCN-01 and HU to induce pathological replication stress (Fig. 3A). Consistently with results from RNAi experiments, MUS81-independent DSBs were observed after treatment in SLX4Δ FA-P cells (Fig. 3B). Interestingly, depletion of MUS81 reduced the amount of DSBs in cells complemented with wild-type SLX4 (WT) while DSBs were not observed in SLX4ΔSAP cells, in which interaction between MUS81 and SLX4 is impaired (Fig. 3B). These results indicate that the presence of SLX4 may actively prevent formation of GEN1-dependent DSBs. Consistently, depletion of GEN1 in SLX4Δ cells suppressed formation of DSBs upon pathological replication arrest (Suppl. Fig. 3A,B). In contrast, treatment with HU for 24 h did stimulate formation of DSBs but their accumulation was as efficiently suppressed by SLX4-depletion as by MUS81 downregulation (Suppl. Fig. 4A,B), suggesting that GEN1 was not involved in processing stalled fork in a more physiological condition.

MUS81 and GEN1 have different substrate specificity *in vitro*, and we wondered whether the protective function of SLX4 against GEN1-dependent DSBs in absence of MUS81 might correlate with formation of a poor GEN1 substrate at demised forks. Indeed, SLX4 through association with SLX1 could stimulate conversion of an intact four-way junction formed upon fork reversal into a nicked four-way junction, which is a good substrate of MUS81 but not of GEN1^3^. Alternatively, SLX4 could hinder GEN1 access to the substrate or prevent transition from an undesirable GEN1 substrate to a more favourable one.

To discriminate between these two hypotheses, we analysed formation of DSBs after pathological replication arrest using the SLX4Δ FA-P cells complemented with wild-type SLX4 (WT), or an SLX4 deletion-mutant abrogating interaction with SLX1 (SLX4ΔSBD)^23^. These cells were transfected with MUS81 siRNAs (Fig. 3C) and treated with UCN-01 and HU to induce pathological replication stress and DSBs. As shown in Fig. 3D, the amount of DSBs after replication fork demise was not significantly affected by the mutation disrupting the interaction of SLX4 with SLX1 (SLX4ΔSBD). As expected, depletion of MUS81 largely decreased DSBs in WT but not in SLX4Δ cells (Fig. 3A,B). Most importantly, MUS81 down-regulation greatly reduced formation of DSBs in SLX4ΔSBD cells (Fig. 3B). These results indicate that processing of demised forks by SLX4/SLX1 is not important to prevent formation of GEN1-dependent DSBs but that is crucial the presence of SLX4. To test if SLX4 prevented GEN1-dependent DSBs through a sort of substrate shielding, we looked for a protein that would able to bind intact four-way junctions (i.e an HJs; the most likely GEN1 substrate) with high affinity. We selected the bacterial RuvA protein, which avidly binds to HJs but does not cleave them^24^. We reasoned that if SLX4 acted through masking or protection of the substrate then overexpression of RuvA would have suppressed formation of DSBs in SLX4Δ cells, also giving us insight into the nature of the substrate cleaved by GEN1 at demised replication forks. To ectopically express RuvA in mammalian cells, the RuvA ORF was cloned in frame with a C-terminal GFP-tag and the SV40 nuclear localisation signal. Transient transfection of the RuvA- GFP-NLS construct into HEK293T cells confirmed expression and nuclear localisation of the RuvA-GFP fusion protein (Suppl. Fig. 5A). Moreover, Western blotting analysis of chromatin fractions from HEK293T cells showed that RuvA-GFP is chromatin associated and that treatment with high doses of CPT, which triggers homology-directed repair, increases its chromatin association (Suppl. Fig. 5B).

**Figure 4:**
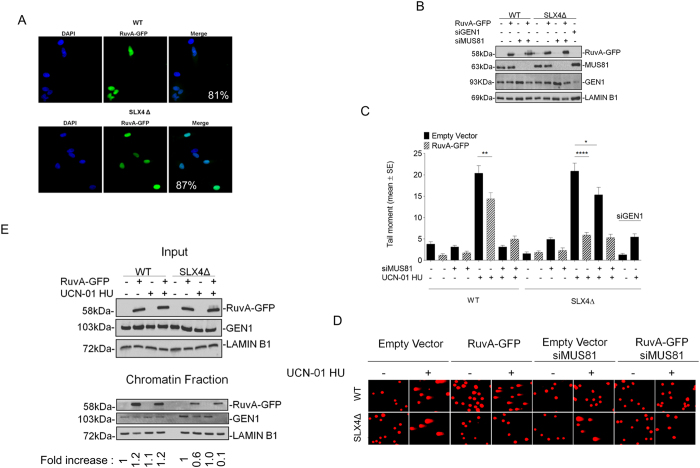
RuvA-GFP expression abrogates GEN1-dependent DSBs in SLX4-deficient FA-P cells. (**A**) Expression of RuvA-GFP tagged protein in SLX4-deficient FA-P cells (SLX4Δ), complemented or not with wild-type SLX4 (WT). Seventy-two hours after transfection, cells were fixed and nuclei were stained with DAPI. Representative microscopy fields are presented. The percentage of RuvA-positive cells in the population is reported in the merged images. (**B**) Western immunoblotting showing RuvA-GFP expression, MUS81 or GEN1 depletion in SLX4-deficient FA-P cells (SLX4Δ), and in cells complemented with wild-type SLX4 (WT). Lysates were prepared 48 h after transfection with indicated plasmid or siRNA oligos. LAMIN B1 was used as a loading control. (**C**) Analysis of DSBs by neutral Comet assay. SLX4-deficient FA-P cells, complemented or not with wild type SLX4, were transfected with RuvA-GFP in combination or not with siRNAs directed against MUS81 (siMUS81) or GEN1 (siGEN1), as indicated. Seventy-two hours after transfections, cells treated for 6 h with 600 nM UCN-01 and 2 mM HU. Graph shows the results from three independent experiments. Data are presented as mean +/− SE. (ns = not significant p > 0.05; *p < 0.05; **p < 0.01; ****p < 0.001, Kruskal-Wallis test). Representative images of comets are presented in (**D**). (**E**) Analysis of GEN1 recruitment in chromatin after replication stress. FA-P cells complemented or not with wild type SLX4 were transfected with RuvA-GFP DNA and GEN1 recruitment in chromatin was evaluated after 6 h of treatments with 600 nM UCN-01 and 2 mM HU by cellular fractionation and Western blotting with indicated antibodies. Before cellular fractionation, an aliquot (10%) of cell suspension was used to assess that amount of GEN1 in all samples (Input). LAMIN B1 was used as a loading control. The fold increase value indicating the amount of GEN1 in chromatin compared to untreated condition is reported below the blot. Uncropped versions of gels are provided in Supplementary Material.

Next, we transfected the empty GFP-NLS plasmid or the RuvA-GFP-NLS construct in the WT or the SLX4Δ cells, and analysed the formation of DSBs after pathological replication arrest induced by combined treatment with UCN-01 and HU. As shown in Fig. 4A,B, RuvA-GFP was efficiently expressed in both WT and SLX4Δ cells, and was nuclear-localized in more than 70% of the cells. As expected, depletion of MUS81 by RNAi (Fig. 4B), greatly prevented formation of DSBs in WT but not in SLX4Δ cells (Fig. 4C,D). Ectopic expression of RuvA-GFP in SLX4Δ cells suppressed DSBs as efficiently as GEN1-depletion, however, it failed to significantly affect formation of DSBs in WT cells after treatment (Fig. 4C,D). To provide clue on competition between RuvA and GEN1 after replication fork demise, we analysed chromatin recruitment of GEN1 in WT and SLX4Δ cells by biochemical fractionation after transfection with the empty vector or the RuvA-GFP plasmid. As shown in Fig. 4E, the amount of chromatin-associated GEN1 is increased in in SLX4Δ cells as compared to the WT but was not altered by the combined UCN-01 and HU treatment. Interestingly, RuvA-GFP expression did not affect levels of chromatin-associated GEN1 in WT cells, irrespective of treatment, but it reduced the amount of GEN1 in chromatin in SLX4Δ cells, especially after treatment with UCN-01 and HU (Fig. 4E).

Collectively, these results indicate that the presence of SLX4 is sufficient to prevent GEN1-dependent DSBs at demised replication forks when MUS81 is absent. Moreover, as ectopic expression of an HJ-binding protein rescues the loss of SLX4-mediated fork protection, our findings suggest that SLX4 hinders access to substrate or prevent the formation of the GEN1 substrate when MUS81 function is compromised.

### GEN1-dependent DSBs take place downstream RAD52 and independently of RAD51 at demised replication forks

**Figure 5:**
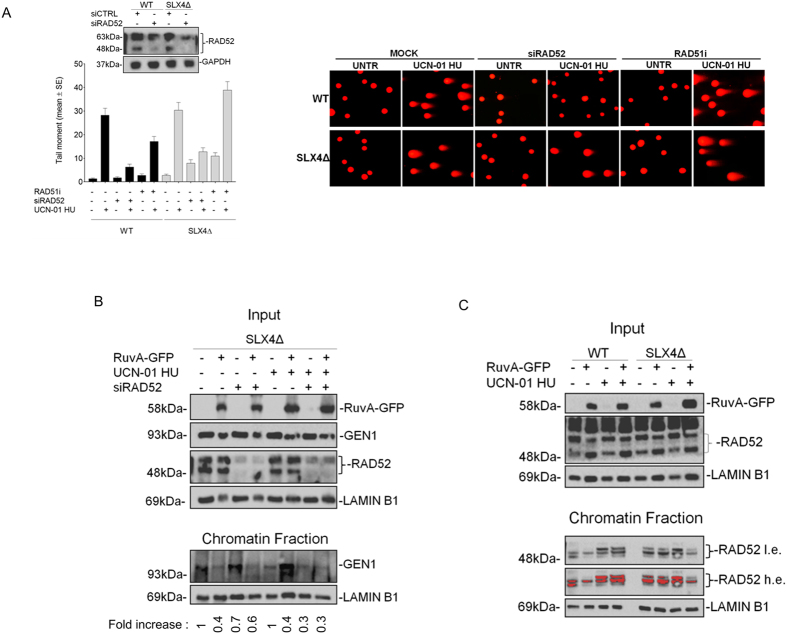
Formation of MUS81 or GEN1-dependent DSBs occurs downstream RAD52 upon replication stress. (**A**) FA-P cells expressing or not wild-type SLX4 were transfected with control siRNA (siCTRL) siRNAs against RAD52 (siRAD52) or treated with a RAD51 inhibitor (RAD51i) before being analyzed for the presence of DSBs by neutral Comet assay. The blot shows actual depletion of RAD52. Results of DSBs formation are presented in the graph as mean tail moment +/− SE from three independent experiments. Representative images are shown in the panel. (**B**) Analysis of GEN1 association with chromatin. SLX4-deficient FA-P cells were treated as indicated and the presence of GEN1 in chromatin evaluated in unfractionated (Input) or fractionated cell extracts (Chromatin Fraction) by Western blotting using the indicated antibodies. LAMIN B1 was used as a loading control. The fold increase value indicating the amount of GEN1 in chromatin compared to untreated condition is reported below the blot. (**C**) Analysis of RAD52 association with chromatin in the presence or not of RuvA. FA-P cells expressing or not wild-type SLX4 were treated as indicated. The presence of RAD52 in chromatin was evaluated in unfractionated (Input) or fractionated cell extracts (Chromatin Fraction) by Western blotting using the indicated antibodies. LAMIN B1 was used as a loading control. (l.e. = low exposure; h.e. = high exposure). Saturated signals are in red. Uncropped versions of gels are provided in Supplementary Material.

We previously reported that formation of DSBs by MUS81 occurs downstream RAD52 but independently of RAD51 after checkpoint inhibition^9^,^25^. However, evidence that GEN1 takes over MUS81 in absence of SLX4, and that RuvA - a bona-fide HJ-binding protein - counteracts such event, prompted us to investigate whether loss of SLX4 would induce a pathway switch in the formation of the GEN1 substrate. Therefore, using WT or SLX4Δ cells, we depleted RAD52 by RNAi or inhibited the RAD51 strand-exchange activity using a small-molecule inhibitor^26^, and analysed formation of DSBs after treatment with UCN-01 and HU. As expected, depletion of RAD52, but not inhibition of RAD51, substantially decreased DSBs at demised replication forks in WT cells (Fig. 5A). Similarly, RAD52 depletion reverted accumulation of DSBs also in SLX4Δ cells, while no significant decrease was observed after treatment with the RAD51 inhibitor (Fig. 5A). As RAD52 depletion prevented accumulation of GEN1-dependent DSBs in SLX4Δ cells, we analysed if chromatin recruitment of GEN1 could be affected by the absence of RAD52 as occurs after ectopic RuvA-GFP expression (Fig. 4E). Hence, we transfected SLX4Δ cells with the RuvA-GFP expression plasmid, RAD52 siRNAs, or both and analysed the presence of GEN1 in chromatin 48 h later upon induction of pathological replication stress. The presence of GEN1 in chromatin was reduced by either ectopic RuvA expression, as expected, or RAD52 depletion, and the effect was more pronounced in cells experiencing replication fork demise (Fig. 5B). Then, we analysed whether the presence of RuvA could alter chromatin recruitment of RAD52. Thus, we performed cellular fractionation experiments in WT and SLX4Δ cells, transfected or not with the RuvA-GFP-NLS plasmid. In agreement with our previous observations^9^, the combined treatment with UCN-01 and HU led to increased association of RAD52 with chromatin in WT cells, and in the appearance of a slowly-migrating form of the protein (Fig. 5C). The amount of chromatin-bound RAD52, and that of its slowly-migrating form, increased in absence of SLX4 already under unperturbed condition and was enhanced further by treatment (Fig. 5C). Interestingly, expression of RuvA did not affect the binding of RAD52 to chromatin in WT cells, however, it slightly reduced chromatin recruitment and appearance of the slowly-migrating of RAD52 after treatment in SLX4Δ cells (Fig. 5C).

Altogether, these findings indicate that GEN1-dependent cleavage occurs downstream RAD52 but independently of RAD51. They also demonstrate that RAD52 depletion or ectopic RuvA expression interferes with GEN1 chromatin association, suggesting that the HJ-like intermediate cleaved by GEN1 may be formed downstream RAD52.

### Late GEN1 function supports viability while GEN1-dependent DSBs at replication forks increase cell death and chromosome instability in SLX4-deficient cells

**Figure 6:**
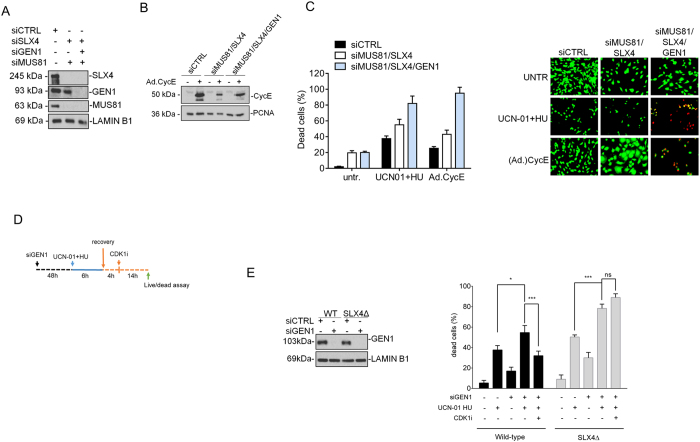
Depletion of GEN1 leads to elevated cell-death before mitosis after replication stress. (**A**) hTERT immortalized fibroblasts were co-transfected with siRNA directed against MUS81 (siMUS81) and SLX4 (siSLX4) in combination or not with siRNAs against GEN1 (siGEN1). Western blotting was used to verify MUS81, SLX4 or GEN1 depletion. LAMIN B1 was used as loading control. (**B**) Evaluation of CycE overexpression by Western blotting. Cells were lysed 72 h after infection with Ad.CycE and lysates analysed by Western blotting using the indicated antibodies. (**C**) Evaluation of cell death in cells depleted of MUS81 and SLX4 alone or in combination with GEN1. After transfection, hTERT immortalized fibroblasts were infected, when indicated, with Ad-CycE at 10 MOI or treated with 600 nM UCN-01 in combination with 2 mM HU to induce replication stress. After treatments, cells were recovered in drug-free medium for 18 h and the number of dead cells evaluated by the LIVE/DEAD assay as described in “Materials and Methods”. Cells stained green are viable while those stained reds are dead. Data are presented as mean percentage of dead cells +/− SE from three independent experiments. Representative images are reported in the panel. (**D**) Experimental scheme used to assess if cell death induced by loss of GEN1 occurs in mitosis. (**E**) Evaluation of cell death induced by replication stress in FA-P-derived cells depleted of GEN1 and arrested in late-G2. Cell death was evaluated by LIVE/DEAD assay as described in “Materials and Methods”. Data are presented as mean percentage of dead cells +/− SE from three independent experiments. Western blotting analysis showing depletion of GEN1 in SLX4-deficient FA-P cells complemented or not with the wild-type SLX4 is provided. (ns = not significant p > 0.05; *p < 0.05, ***p < 0.001, Mann-Whitney test). Uncropped versions of gels are provided in Supplementary Material.

Cells depleted of MUS81 or SLX4 are hypersensitive to replication stress induced by inhibition of CHK1 and HU treatment^9^. Since we found that GEN1 replaces MUS81 to form DSBs in SLX4-depleted cells after oncogene-induced replication stress or checkpoint inhibition, we asked whether GEN1-dependent DSBs in S-phase were required for proliferation after pathological replication stress. To this end, we co-depleted SLX4 and MUS81, which participate in a common pathway, alone or in combination with GEN1 in hTERT-immortalised normal fibroblasts and analysed cell death after recovery from a 6 h treatment with UCN-01+HU or upon CycE overexpression (Fig. 6A,B). As shown in Fig. 6C, co-depletion of SLX4 and MUS81 enhanced cell death already under unperturbed cell growth and GEN1 knock-down did not increase further cell death. Loss of SLX4 and MUS81 sensitized cells to pathological replication stress, although the effect was more evident after the combined UCN-01 and HU treatment (Fig. 6C). Of note, concomitant depletion of GEN1, SLX4 and MUS81 essentially resulted in almost complete cell death after treatments (80–90%).

Next, we assessed whether the enhanced cell death observed upon depletion of GEN1 in SLX4-deficient cells occurred during mitosis because of unresolved recombination intermediates formed consequently to replication fork demise. To this end, WT and SLX4Δ cells were depleted or not of GEN by RNAi, challenged with UCN-01 and HU to induce pathological replication stress, and recovered in the presence or not of the CDK1 inhibitor RO-3306 to prevent G2-M transit (Fig. 6D).

Depletion of GEN1 enhanced cell death in WT and SLX4Δ cells, although the effect in absence of SLX4 was more pronounced (Fig. 6E). After treatment, impaired G2-M transition reduced cell death of about 50% in WT cells, counteracting GEN1 depletion, but not in SLX4Δ cells (Fig. 6E). These results suggest that, while in WT cells the effect on viability associated with depletion of GEN1 derives from progression into mitosis with unresolved intermediates, in SLX4Δ cells the elevated cell death associated with loss of GEN1 does not.

**Figure 7:**
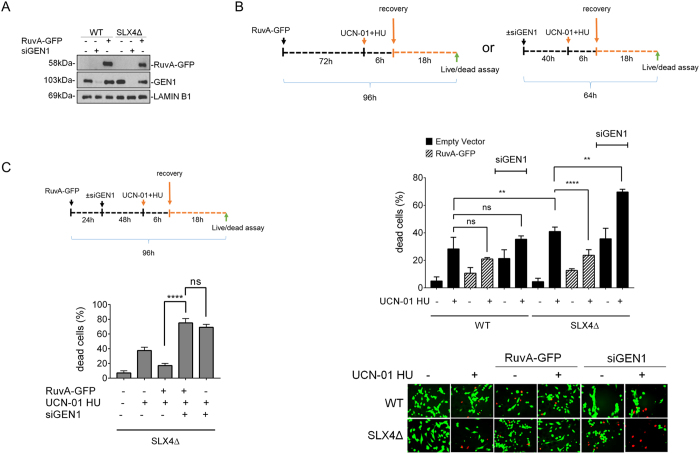
RuvA expression increases viability of FA-P cells after pathological replication stress. (**A**) Analysis of RuvA-GFP expression or GEN1 depletion by Western blotting in FA-P and FA-P complemented cells. Immunoblotting was performed using the indicated antibodies. GAPDH was used as loading control. (**B**) After transfection with RuvA-GFP transfection or siRNAs oligo, as depicted in the experimental scheme, cells were treated with UCN-01 (600 nM) and HU (2 mM) and analyzed by LIVE/DEAD assay. Data are presented as percentage of dead cells and are mean values from three independent experiments. Error bars represent standard errors (SE). (ns = not significant p > 0.05; **p < 0.01, ****p < 0.0001, Mann-Whitney test). The panel shows representative images: live cells are green stained, while dead cells are red. (**C**) GEN1 depletion counteracts RuvA expression. Twenty-four hours after transfection with RuvA-GFP, FA-P cells were transfected with GEN1 siRNAs (siGEN1) and 24 h thereafter cells were treated with UCN-01 (600 nM) and HU (2 mM) for 6 h, according to the experimental scheme. After the recovery, cells were analyzed by LIVE/ DEAD assay. Data are presented as percentage of dead cells and are mean values from three independent experiments. Error bars represent standard errors. (ns = not significant p > 0.05; **p < 0.01, ****p < 0.0001, Mann-Whitney test). Uncropped versions of gels are provided in Supplementary Material.

Having established that ectopic RuvA expression interferes with formation of GEN1-dependent DSBs occurring in S-phase when SLX4 is absent, we further explored the effect on cell viability of impaired GEN1-dependent cleavage by transfecting the RuvA-GFP-NLS plasmid in WT and SLX4Δ cells (Fig. 7A). As expected, absence of SLX4 sensitized cells to pathological replication arrest induced by UCN-01 and HU (Fig. 7B). Surprisingly, however, although ectopic expression of RuvA slightly increased cell death in untreated cells, it significantly rescued viability of SLX4Δ cells after treatment (Fig. 7B). Of note, ectopic RuvA expression also largely rescued the prominent S-phase arrest observed in SLX4Δ cells because of pathological replication stress, while having a less dramatic effect on WT cells (Suppl. Fig. 6).

The main physiological role of GEN1 is the resolution of recombination intermediates in mitosis^22^. Thus, we wondered whether the discrepancy between the effect on the viability of GEN1 depletion and RuvA expression could derive from a dual requirement of GEN1. We reasoned that rescue of viability observed in SLX4Δ cells after ectopic RuvA expression could derive from a regained availability of substrates to GEN1 later on during recovery, when ectopic RuvA protein level was substantially reduced (Suppl. Fig. 7). To test this possibility, we down-regulated GEN1 in SLX4Δ cells, in which RuvA-GFP-NLS has been transfected 24 h before, and analysed cell death after recovery from the UCN-01+HU treatment (Fig. 7C). Interestingly, depletion of GEN1, even in the presence of ectopic RuvA, was sufficient to induce cell death in SLX4Δ cells at a level comparable to that of the same cells transfected with only GEN1 siRNA (Fig. 7C).

Altogether, our results indicate that the reduced viability observed after replication fork demise in absence of SLX4 and GEN1 is mostly the consequence of loss of GEN1 activity during recovery. In contrast, interfering only with GEN1-dependent processing of demised forks in S-phase is beneficial to viability of SLX4-deficient cells.

### Unscheduled activation of GEN1 is responsible for genome instability associated with loss of SLX4

**Figure 8:**
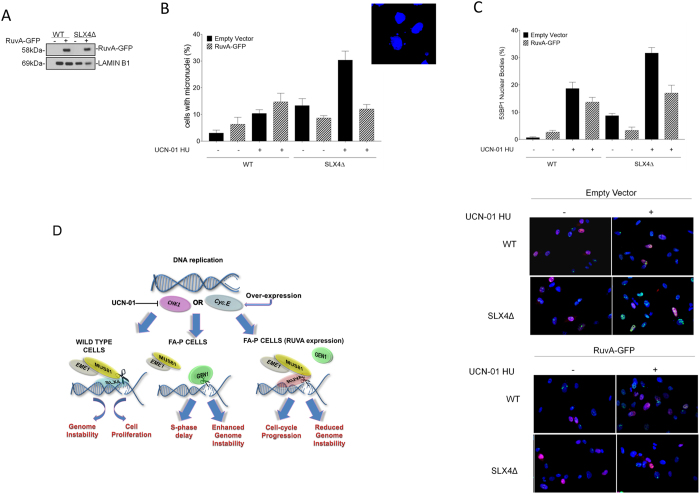
GEN1 premature activation causes genome instability in absence of SLX4. (**A**) Western blotting analysis showing expression of RuvA-GFP in FA-P-derived cells. (**B**) Analysis of micronuclei (MN). Forty-eight hours after RuvA-GFP transfection, FA-P and FA-P complemented cells were treated with 600 nM UCN-01 and 2 mM HU for 6 h, recovered overnight in drug-free medium and analysed for the presence of MN by DAPI staining. Data are presented as mean values of percentage of cells with MN from three independent experiments. Error bars represent standard errors. The panel shows representative images of micronuclei-containing FA-P cells after replication stress. (ns = not significant p > 0.05; **p < 0.01, ****p < 0.0001, Mann-Whitney test). (**C**) 53BP1 NBs formation in FA-P and FA-P complemented cells. After RuvA-GFP transfection, cells were treated with 600 nM UCN-01 and 2 mM HU for 6 h, recovered overnight in drug-free medium and analysed for the presence of 53BP1 NBs in G1 cells by co-immunofluorescence using anti-Cyclin-A and anti-53BP1 antibodies. The presence of 53BP1-positive G1 cells (Cyclin-A negative) for each condition was reported in the graph as mean +/− SE. from three independent experiments. (ns = not significant p > 0.05; **p < 0.01, ***p < 0.001, ****p < 0.0001, Mann-Whitney test). Representative images are presented in the panels. (**D**) Cartoon summarizing our results. Under pathological conditions, as induced by CycE overexpression or CHK1 inhibition, recruitment of MUS81-EME1 complex by SLX4 causes DSBs. Repair of DSBs can lead to genome instability but ensures cell proliferation. Only when SLX4 is absent, as occurs in FA-P cells, GEN1 takes over MUS81-EME1 complex. However, unscheduled GEN1 activation in S-phase forms DSBs increasing much more genome instability and undermining cell survival. If RuvA expression interferes with GEN1 cleavage in S-phase it can rescue genome stability and cellular proliferation (see Discussion for details). Uncropped versions of gels are provided in Supplementary Material.

Loss of SLX4 greatly affects genome integrity of cells^17^,^20^. Our data suggest that GEN1-dependent DSBs formed in SLX4-deficient S-phase cells after replication stress support replication but also undermines viability. Thus, we investigated if increased genome instability of SLX4Δ cells was related to processing of demised replication forks by GEN1. To this end, we evaluated formation of micronuclei (MN) and 53BP1 nuclear bodies (53BP1 NBs), two acknowledged readouts of genome instability found elevated in cells depleted of SLX4^17^. To prevent GEN1 from targeting intermediates at replication forks, RuvA was expressed before treating cell with UCN-01 and HU to induce replication fork demise (Fig. 8A). As expected, the percentage of MN increased in absence of SLX4, and pathological replication arrest heightened the phenotype greatly (Fig. 8B). Interestingly, RuvA expression enhanced the frequency of MN in unperturbed WT cells but failed to affect their formation after treatment. In contrast, ectopic RuvA expression significantly reduced the percentage of MN in untreated SLX4Δ cells, and this reduction was more striking upon replication fork demise (Fig. 8B).

Consistently, the percentage of 53BP1 NBs in G1-phase (i.e. Cyclin A-negative cells) increased in absence of SLX4 (Fig. 8C). Moreover, treatment with UCN-01+HU raised the number of 53BP1 NBs-positive cells of more than two-fold in SLX4Δ cells, while only slightly elevated their formation in WT cells (Fig. 8C). Strikingly, and similarly to what obtained in the analysis of MN, ectopic RuvA expression rescued the increase of 53BP1 NBs-positive cells associated with loss of SLX4 almost completely (Fig. 8C). Of note, the opposite effect (i.e. an increase in 53BP1 NBs) was associated to depletion of GEN1 in SLX4Δ cells (Suppl. Fig. 7). As ectopic RuvA expression did not prevent progression to M-phase after recovery from replication fork demise (Suppl. Fig. 8), the observed reduced genome instability was unlikely to be a cell cycle-related effect.

Collectively, our findings indicate that GEN1-dependent DSBs arising at demised replication forks in absence of SLX4 undermine genome integrity, suggesting that at least a fraction, if not the majority, of genome instability associated with loss of SLX4 derives from unprogrammed function of GEN1.

## Discussion

Several lines of evidence indicate that SLX4 is crucial to direct different nuclease at the site of action during resolution of recombination intermediates^12^,^13^,^27^,^28^,^29^,^30^. Although recombination intermediates may be targeted by multiple nucleases^3^,^31^, little is known about what happens at demised replication forks. In response to checkpoint inhibition or oncogene activation, perturbed replication forks collapse and degenerate into DSBs^32^. Formation of DSBs at perturbed forks by the MUS81 complex contributes to viability under replication stress, although it generates genomic instability^8^,^9^. However, under pathological replication stress, DSBs are still observed in absence of SLX4^9^. Here, we show that SLX4 is indeed necessary to support MUS81 function at demised replication forks, even if this crucial role is masked by the take-over of GEN1-dependent DSBs. In late G2-phase and in mitosis, loss of MUS81 or SLX4 is mitigated by GEN1, which represents a salvage pathway in yeast and human cells^16^,^17^,^20^,^28^,^33^. We previously described that GEN1-dependent DSBs are observed in S-phase only if RAD52 and MUS81 are co-depleted^9^. Now, our data indicate that formation of GEN1-dependent DSBs in S-phase might occur under other pathological conditions. GEN1-dependent processing observed upon depletion of MUS81 and RAD52 occurs because of a RAD52-RAD51 switch that most likely results in the formation of a GEN1-cleavable substrate (i.e. an intact HJ), as evidenced by the different genetic dependencies of the two processes^9^. In contrast, GEN1-dependent DSBs observed in absence of SLX4 maintain the same genetic dependency on RAD52 as that formed by MUS81. Thus, our finding would support the notion that GEN1 may act as a SLX4-MUS81 back-up pathway independently of the pathway by which its substrate is formed. The identity of the intermediate targeted by the MUS81 complex at demised replication forks is still uncertain. The genetic dependency on RAD52 and the striking ability to process D-loops assembled *in vitro* through the RAD52 annealing activity, suggested that, upon CHK1 inhibition, MUS81 complex may target D-loops generated by fork reversal and subsequent invasion of nascent strand back in the template^9^. However, electron microscopy analysis evidenced that the MUS81 complex may cleave reversed forks, at least in cells overexpressing oncogenic CDC25^10^. In both cases, also given the clear MUS81 preference towards nicked HJ, the intermediate formed at demised replication forks would be not easily targeted by GEN1. Indeed, although *in vitro* GEN1 can process forked DNA structures, it is considered as a true HJ resolvase *in vivo*^22^,^34^,^35^. Thus, GEN1-dependent processing would require further remodelling at the fork. For instance, an unprocessed D-loop formed at a demised replication fork might generate an intact HJ, which could be targeted by GEN1, as it has been proposed in yeast during break-induced replication^36^. As we found that SLX4 is sufficient to prevent GEN1 from taking-over MUS81 at demised replication forks, MUS81-alternative and potentially highly-mutagenic processing of demised forks by GEN1 may be regulated by multiple mechanisms in human cells, in addition to nuclear exclusion^22^. Interestingly, we show that, in absence of SLX4, formation of GEN1-dependent DSBs at demised replication forks can be prevented by ectopic expression of the bacterial RuvA protein. RuvA is a specific HJ-binding protein^24^, and its protective effect on GEN1-dependent DSBs may indicate that an intact HJ is formed at stalled replication forks after checkpoint inhibition only if SLX4 is absent or that HJs form anyway and SLX4 hinders their access to GEN1. Since GEN1-dependent DSBs form downstream RAD52 but independently of RAD51, it is possible that GEN1 targets intact HJs formed upon migration of the D-loop, which cannot be processed by MUS81 in absence of SLX4. Another alternative explanation may be that ectopic RuvA expression leads to freezing of regressed forks from which RAD52-dependent D-loops originate. In this scenario, binding of the regressed fork by RuvA should also prevent formation of DSBs in wild-type cells because would interfere with formation of the MUS81-complex substrate. However, this is unlikely as RuvA expression does not revert MUS81-dependent DSBs in wild-type cells. Of note, in SLX4Δ cells, GEN1-dependent DSBs are still prevented by expression of an SLX4 deletion mutant that is unable to bind SLX1. From one hand, the irrelevance of SLX1 indicates that it is not the ability to induce an SLX1-mediated nick in an HJ-like structure that contributes to the SLX4-dependent inhibition of fork processing by GEN1. From the other hand, this would be consistent with the reported unnecessary role of SLX1 for the SLX4-MUS81 function in S-phase^17^.

Our data implicate that GEN1-dependent DSBs accumulate even in the presence of an active BLM-TopIII-RMI1/2 complex (BTR). This finding suggests that dHJs do not form in SLX4-deficient cells upon CHK1 inhibition, or that, as described previously, CHK1 inhibition interferes with BLM functionality^37^,^38^. In yeast and human cells, GEN1 is synthetic lethal with SLX4-MUS81 after DNA damage or even under unperturbed cell growth^17^,^20^,^31^,^33^. Our data expand this genetic relationship to pathological replication stress and indicate that loss of viability associated with SLX4 and GEN1 deficiency is not the consequence of impaired processing at demised forks in S-phase but rather the result of poor resolution before mitosis. Indeed, viability of SLX4-deficient cells under pathological replication stress is improved if GEN1-dependent DSBs in S-phase are prevented by ectopic expression of RuvA and concomitantly GEN1 function is recovered in G2/M phase. This observation would be consistent with proliferation being most affected by loss of resolution of late recombination intermediates accumulating as a consequence of defective replication^16^,^17^,^20^. Interestingly, SLX4/GEN1-deficient cells undergo cell death before entering mitosis and not upon progression into mitosis with unresolved intermediates, as it occurs in wild-type cells depleted of GEN1.

Although the function of GEN1 has been reported to contribute to fork recovery in SLX4-deficient cells^20^, we observe that its action at demised forks has detrimental effects on genome stability whereas the function of GEN1 at later stages after recovery is important for chromosome integrity. Indeed, ectopic RuvA expression in SLX4-null cells, but not GEN1 depletion altogether, can reduce chromosome damage after CHK1 inhibition. Of note, enhanced formation of 53BP1 NBs in unperturbed SLX4-null cells can be almost completely reverted by interfering with GEN1 function through ectopic RuvA expression. Formation of 53BP1 NBs in G1-phase has been associated to persistence of unreplicated or unresolved DNA regions at common fragile sites, which can contribute to the intrinsic genome instability of FA-P cells^18^,^19^,^23^.

Altogether, these findings suggest a model to illustrate the unexpected function of SLX4 in response to pathological replication stress to prevent deleterious cleavage of demised replication forks by GEN1 (Fig. 8D). Moreover, our results corroborate the notion that, at least under pathological replication conditions, such as those typical during cancer development, nuclease activation at demised forks may be detrimental to genome integrity. Interestingly, SLX4 mutations have been associated to Fanconi anaemia, and are found in sporadic tumours as well. Since replication stress is a common feature of cancer, it is tempting to speculate that the unscheduled GEN1 activation in S-phase might explain, at least in part, the genomic instability of these cells. Thus, our study highlights the importance of a fully understanding of how these pathways function and are regulated to improve our knowledge on the origin of genome instability in cancer and to identify better therapeutic approaches.

## Materials and Methods

### Cell lines and culture conditions

The hTERT-immortalized human normal primary fibroblasts GM01604 were obtained from Prof. Shay and Prof. Wright (Southwestern University of Texas) and were described in Ouellette *et al*.^39^. The FA-P cell lines RA3331/E6E7/hTERT and its derivatives complemented with expressing the wild-type, SAP or SBD-deleted form of SLX4 were a kind gift of Prof. Smogorzewska (Rockefeller University of New York)^23^. HEK 293T cells were maintained in Dulbecco’s modified Eagle medium (DMEM) supplemented with 10% fetal bovine serum (FBS). All cultures were grown at 37 °C in a humidified atmosphere containing 10% CO_2_. Cell lines were routinely tested for mycoplasma contamination and maintained in cultures for no more than one month.

### Treatments and RNAi

Hydroxyurea (HU) or MMC was added to culture medium at the indicated concentrations from stock solutions prepared in PBS to induce DNA replication arrest or DNA damage. To inhibit CHK1 activity, we used the UCN-01 compound (ALEXIS biochemicals) at a 600 nM concentration. To accumulate cells in M-phase, nocodazole (Sigma Aldrich) was used at 0,05 μg/ml. RO3306 (Selleck), a CDK1 inhibitor, was prepared in DMSO and added from a stock solution at a concentration of 20 μM. The B02 compound (Selleck), an inhibitor of RAD51 activity, was used at 27 μM. Clorodeoxyuridine (CldU) (Sigma-Aldrich) was dissolved in sterile DMEM as a 200 mM stock solution and used at 20 μM. SLX4, GEN1 and MUS81 expression was knocked-down by transfection with specific siRNAs directed against the coding sequence of the gene (Qiagen) using Interferin siRNAs delivery reagent (Polyplus).

### Western blotting analysis

Western blots were performed using standard methods. Blots were incubated with primary antibodies against: anti-MUS81 (Santa Cruz Biotechnology, 1:2000), anti-SLX4 (Novus Biological, 1:1000), anti-GEN1 (Origene, 1:500), anti-Cyclin-E (Santa Cruz Biotechnology, 1:2000), anti-GFP (Santa Cruz Biotechnology, 1:1000), Lamin B1 (Abcam, 1:10000), anti-RAD52 (Santa Cruz Biotechnology, 1:500), anti-GAPDH (Millipore, 1:5000). After incubations with horseradish peroxidase-linked secondary antibodies (Jackson ImmunoResearch), the blots were developed using the chemiluminescence detection kit ECL-Plus (Amersham) according to the manufacturer’s instructions. Quantification was performed on scanned images of blots using Image Lab software, and values shown on the graphs represent a normalization of the protein content evaluated through Lamin B1 immunoblotting.

### Neutral Comet assay

DNA breakage induction was evaluated by Comet assay (single cell gel electrophoresis) in non-denaturing conditions as described in Murfuni *et al*.^8^. Briefly, dust-free frosted-end microscope slides were kept in methanol overnight to remove fatty residues. Slides were then dipped into molten Low Melting Point (LMP) agarose at 0.5% and left to dry. Cell pellets were resuspended in PBS and kept on ice to inhibit DNA repair. Cell suspensions were rapidly mixed with LMP agarose at 0.5% kept at 37 °C and an aliquot was pipetted onto agarose-covered surface of the slide. Agarose embedded cells were lysed by submerging slides in lysis solution (30 mM EDTA, 0,1% SDS) and incubated at 4 °C, 1 h in the dark. After lysis, slides were washed in TBE 1X running buffer (Tris 90 mM; boric acid 90 mM; EDTA 4 mM) for 1 min. Electrophoresis was performed for 20 min in TBE 1X buffer at 0.5 V/cm. Slides were subsequently washed in distilled H_2_O and finally dehydrated in ice cold methanol. Nuclei were stained with GelRed (1:1000) and visualized with a fluorescence microscope (Zeiss), using a 60X objective, connected to a CCD camera for image acquisition. At least 300 comets per cell line were analyzed using CometAssay IV software (Perceptive instruments) and data from tail moments processed using Prism software. Apoptotic cells (smaller Comethead and extremely larger Comettail) were excluded from the analysis to avoid artificial enhancement of the tail moment.

### Immunofluorescence

Cells were grown in 35-mm coverslips and harvested at the indicated times after treatments. For 53BP1 IF, after further washing with PBS, cells were fixed with 4% PFA at RT for 10 min. Cells were subsequently permeabilized with 0.4% Triton-X100. Staining with mouse polyclonal anti-53BP1 (1:300, Millipore), γ-H2AX (1:1000 Santa Cruz Biotechnology) or rabbit polyclonal anti-Cyclin A (1:100 Santacruz), pS10H3 (1:1000 Santa Cruz Biotechnology) diluted in a 1%BSA/0,1% saponin in PBS solution, was carried out for 1 h at RT. After extensive washing with PBS, specie-specific fluorophore-conjugated antibodies (Invitrogen) were applied for 1 h at RT followed by counterstaining with 0.5 mg/ml DAPI. Secondary antibodies were used at 1:200 dilution. Images were acquired as greyscale files using Metaview software (MDS Analytical Technologies) and processed using Adobe Photoshop CS3 (Adobe). For each time point, at least 200 nuclei were examined and foci were scored at 40×.

### Micronuclei analysis

Micronuclei analysis was performed through immunofluorescence on DAPI-stained nuclei. At the end of treatments, cells were washed two times in PBS, then fixed in PFA 4% in PBS in the dark for 10 min at RT. After two washes with PBS, cells were subjected to permeabilization with Triton X-100 0,4% for 10 min e the washed again with PBS. Staining with DAPI 0,5 μg/ml was carried out for 5 min at RT. After a further wash in PBS, slides were mount and images were randomly acquired with Eclipse 80i Nikon Fluorescence Microscope, equipped with a VideoConfocal (ViCo) system. Acquired images were analyzed by scoring of cells with micronuclei, which were shown in a graph as a percentage of cells with micronuclei (at least 200 nuclei).

### Immunofluorescence staining of DSBs in replication foci

DNA replication sites were visualized by incorporation of CldU into DNA. CldU labelling was added 15 min before treatments. Cells were then fixed with 4% PFA for 10 min at RT. Cells were subsequently permeabilized with 0.4% Triton-X100 and blocked with 10% FBS/PBS for 1 h at RT. Staining with γ-H2AX (1:1000 Santa Cruz Biotechnology) in a 1% BSA/0, 1% saponin in PBS solution was performed for 1 h at RT. After extensive washing with PBS, anti-mouse specific fluorophore-conjugated antibodies (Invitrogen) were applied for 1 h at RT. Cells were postfixed with metOH/Acetic Acid for 20 min at RT, then were treated with HCL 2,5 M for 1 h at RT for DNA denaturation. After extensive washing with PBS, staining with rat anti-CldU/BrdU (1:100 Abcam) in a 1% BSA/0,1% saponin in PBS solution was performed for 1 h at RT. After extensive washing with PBS, specific anti-rat fluorophore-conjugated antibodies (Invitrogen) were applied for 1 h at RT followed by counterstaining with 0.5 mg/ml DAPI. Images were acquired as greyscale files using Metaview software (MDS Analytical Technologies) and processed using Adobe Photoshop CS3 (Adobe). For each time point, at least 200 nuclei were examined and foci were scored at 40×.

### LIVE/DEAD staining

Cell viability was evaluated by the LIVE/DEAD assay (Sigma-Aldrich) according to the manufacturer. Cell number was counted in randomly-chosen fields and expressed as percent of dead cells (number of red nuclear stained cells/total cell number normalized for the dead cells floating). For each time point, at least 200 cells were counted.

### Protein cloning

The RuvA ORF was amplified by colony-PCR using the following primers: PRIMER SEQUENCE FORWARD: 5′-ATTAAGCTTATAGGCAGACTCAGAGGC-3′ and REVERSE: 3′-ATTCTTAAGTATTGCGCCGCGCATC-5′. The ORF of RuvA protein was cloned into a mammalian expression plasmid in frame with an N-terminal GFP-tag and a C-terminal nuclear localisation signal (NLS pEGFP-N3 (1299) was a gift from Eric Schirmer (Addgene plasmid #62043). Cloning was PCR-verified and sequence validated.

### Cell cycle analysis by flow cytometry

Flow cytometric cell cycle analysis was performed using standard procedures. Cell pellets were resuspended, and cells stained with PI solution (propidium iodide 20 μg/ml) for 30 min at 4 °C in the dark. Samples were analyzed by flow cytometry, and data analysis was performed with CellQuest software.

### Statistical analysis

All the data are presented as means of at least three independent experiments. Statistical comparisons of knock-down or mutant cells to their relevant control were analyzed by Mann-Whitney test. P < 0.05 was considered significant. Multiple comparisons between samples were performed by Kruskal-Wallis test. P < 0.05 was considered significant. The number of asterisks denoted the level of significance as follow: ****p < 0.0001; ***p < 0.001; **p < 0.01; *p < 0.05.

## Additional Information

**How to cite this article:** Malacaria, E. *et al*. SLX4 Prevents GEN1-Dependent DSBs During DNA Replication Arrest Under Pathological Conditions in Human Cells. *Sci. Rep.* **7**, 44464; doi: 10.1038/srep44464 (2017).

**Publisher's note:** Springer Nature remains neutral with regard to jurisdictional claims in published maps and institutional affiliations.

## Supplementary Material

Supplementary Figures
