# Supplementary material for: SLX4 Prevents GEN1-Dependent DSBs During DNA Replication Arrest Under Pathological Conditions in Human Cells
Source: Sci Rep. 2017 Mar 14;7:44464. doi: 10.1038/srep44464 (PMC5349550; doi:10.1038/srep44464)
Supplement: Supplementary Figures [file srep44464-s1.pdf]

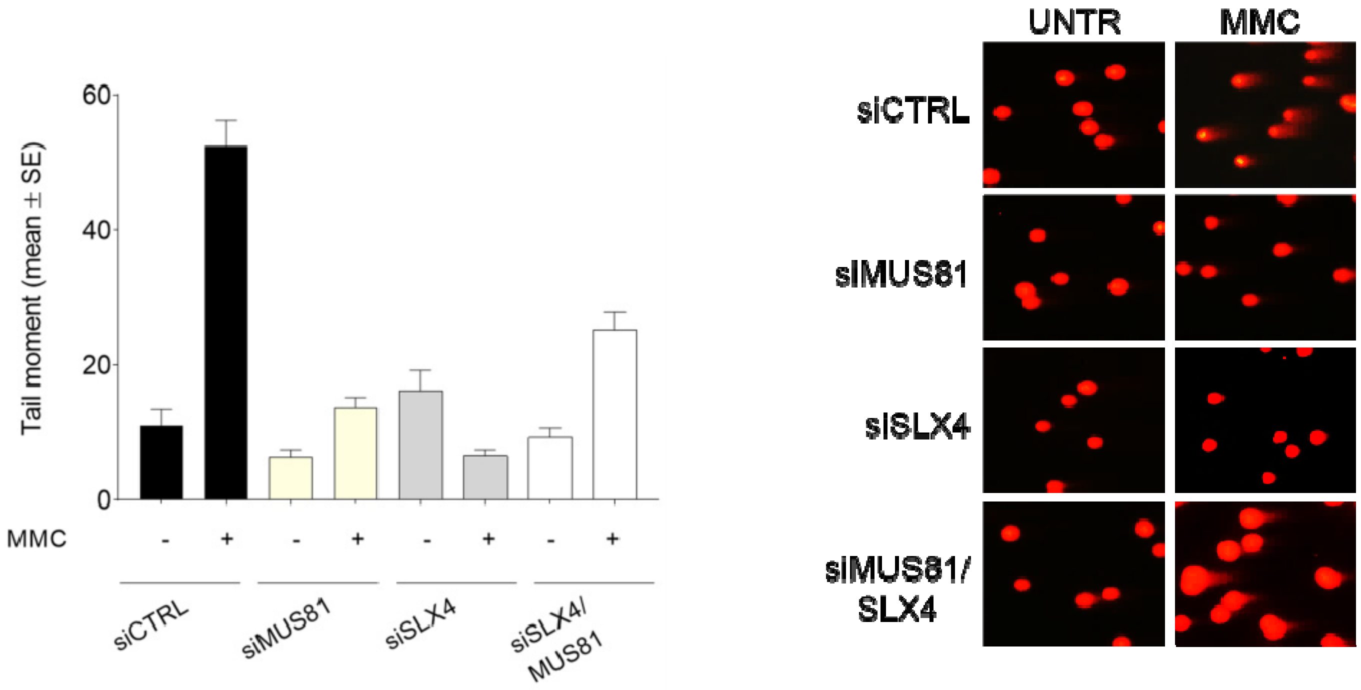

Supplementary Figure 1

A

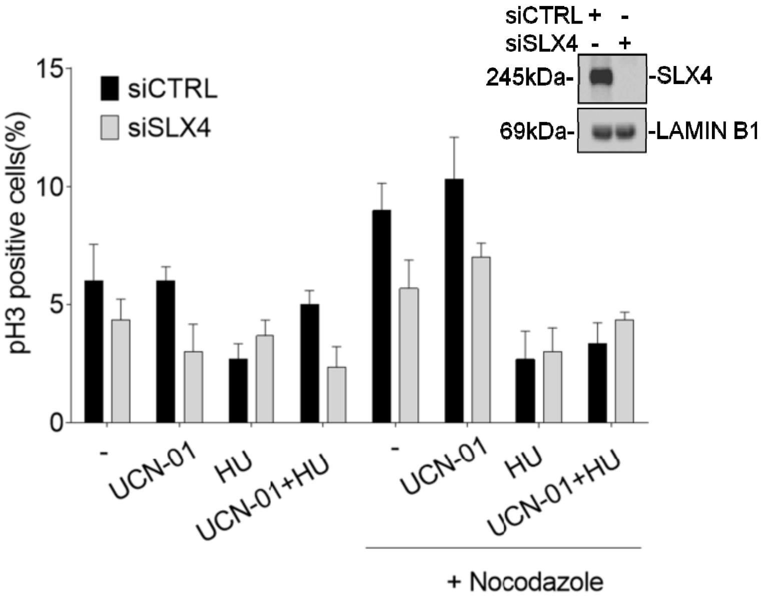

B

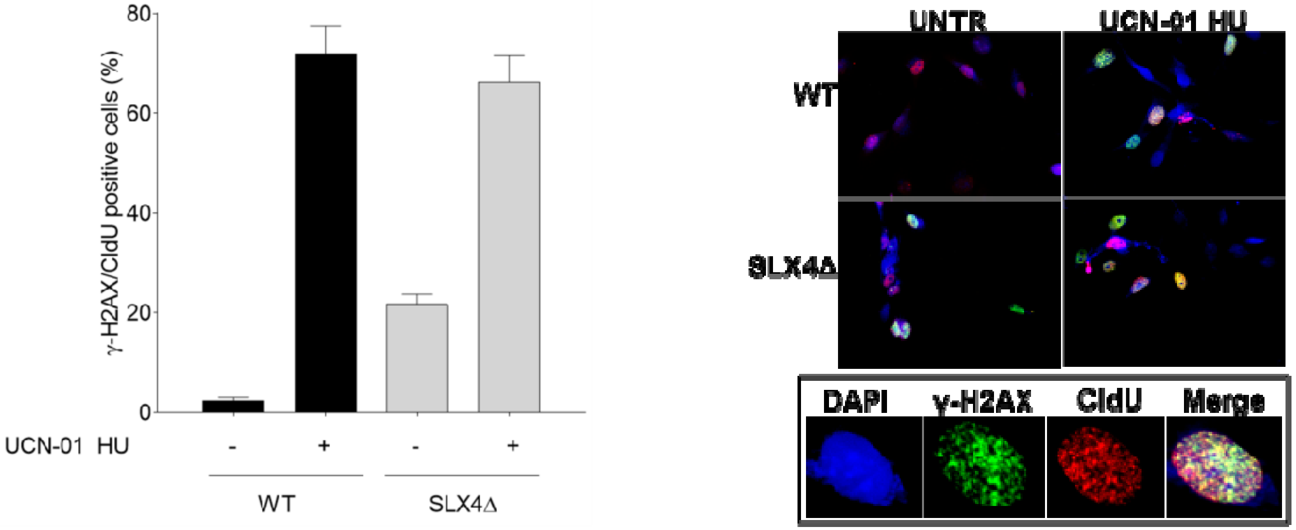

Supplementary Figure 2

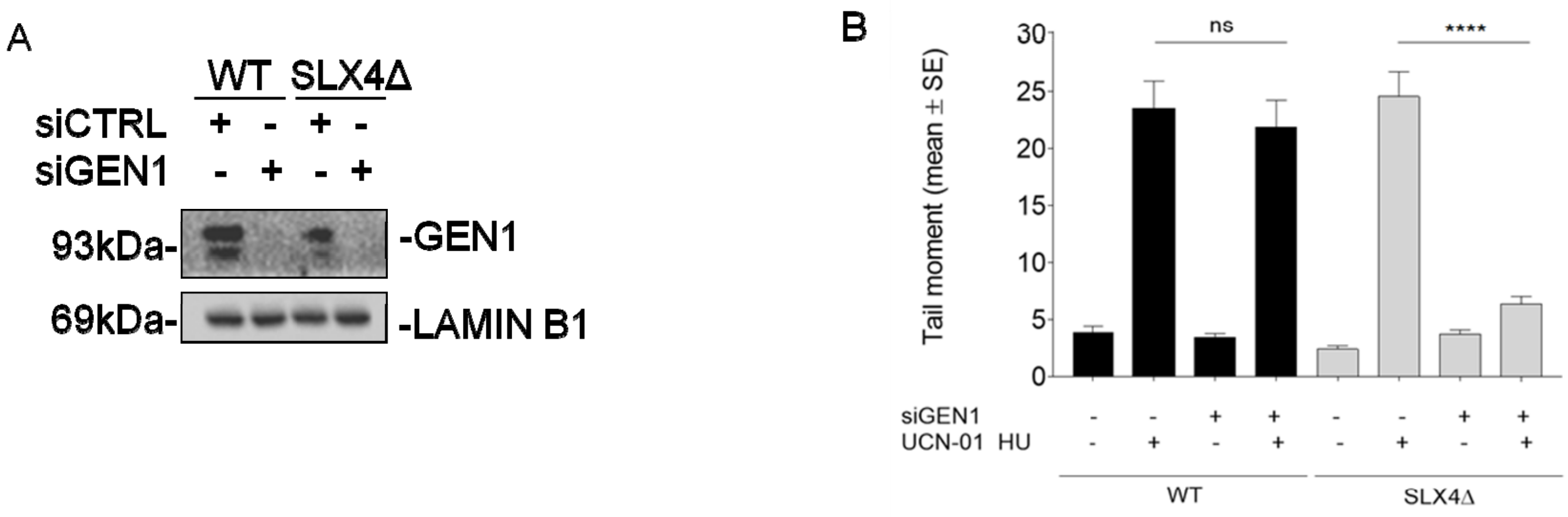

Supplementary Figure 3

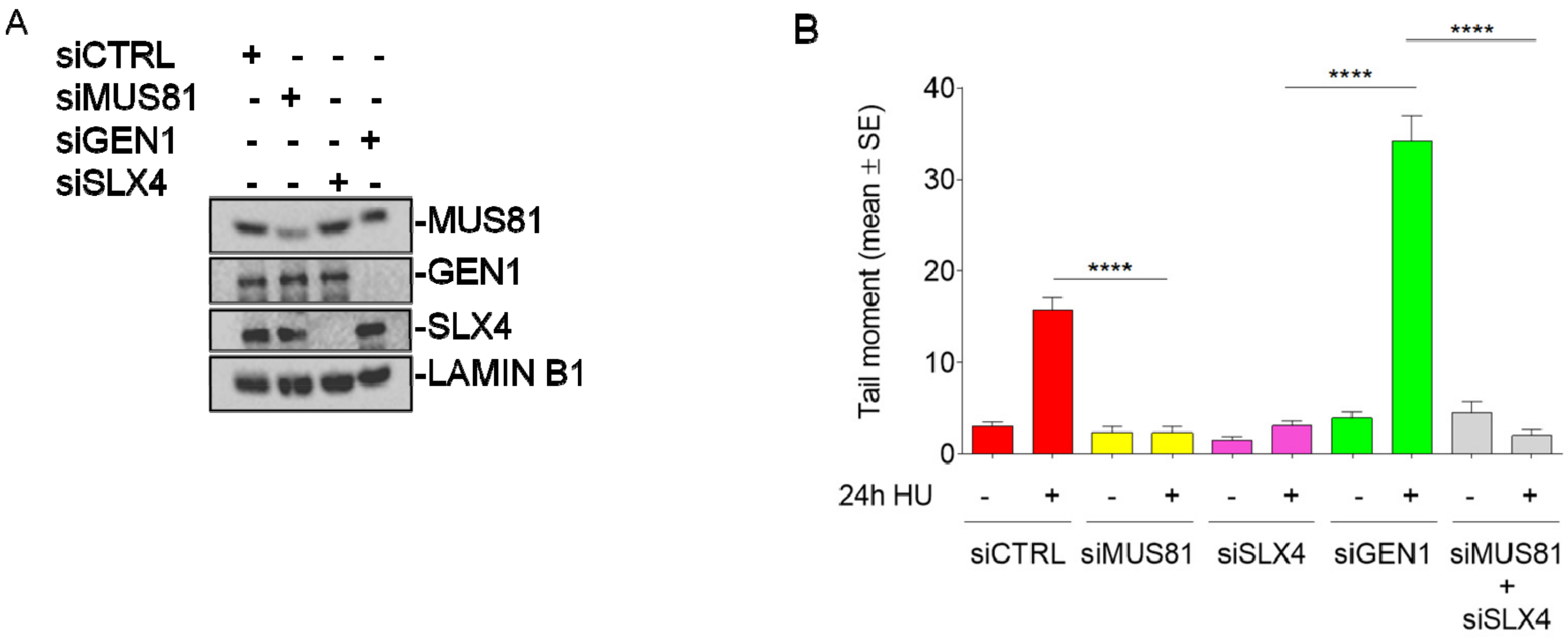

Supplementary Figure 4

A

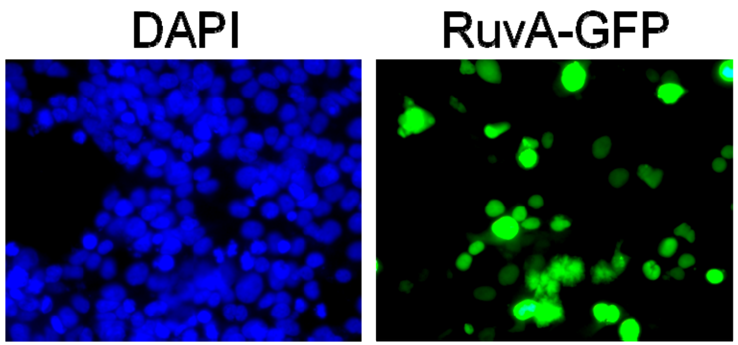

B

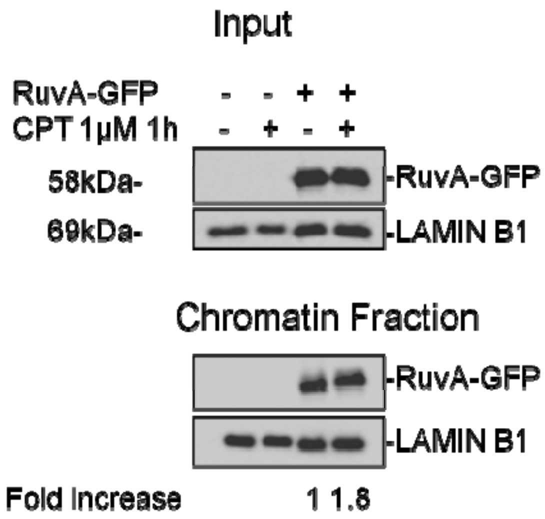

Supplementary Figure 5

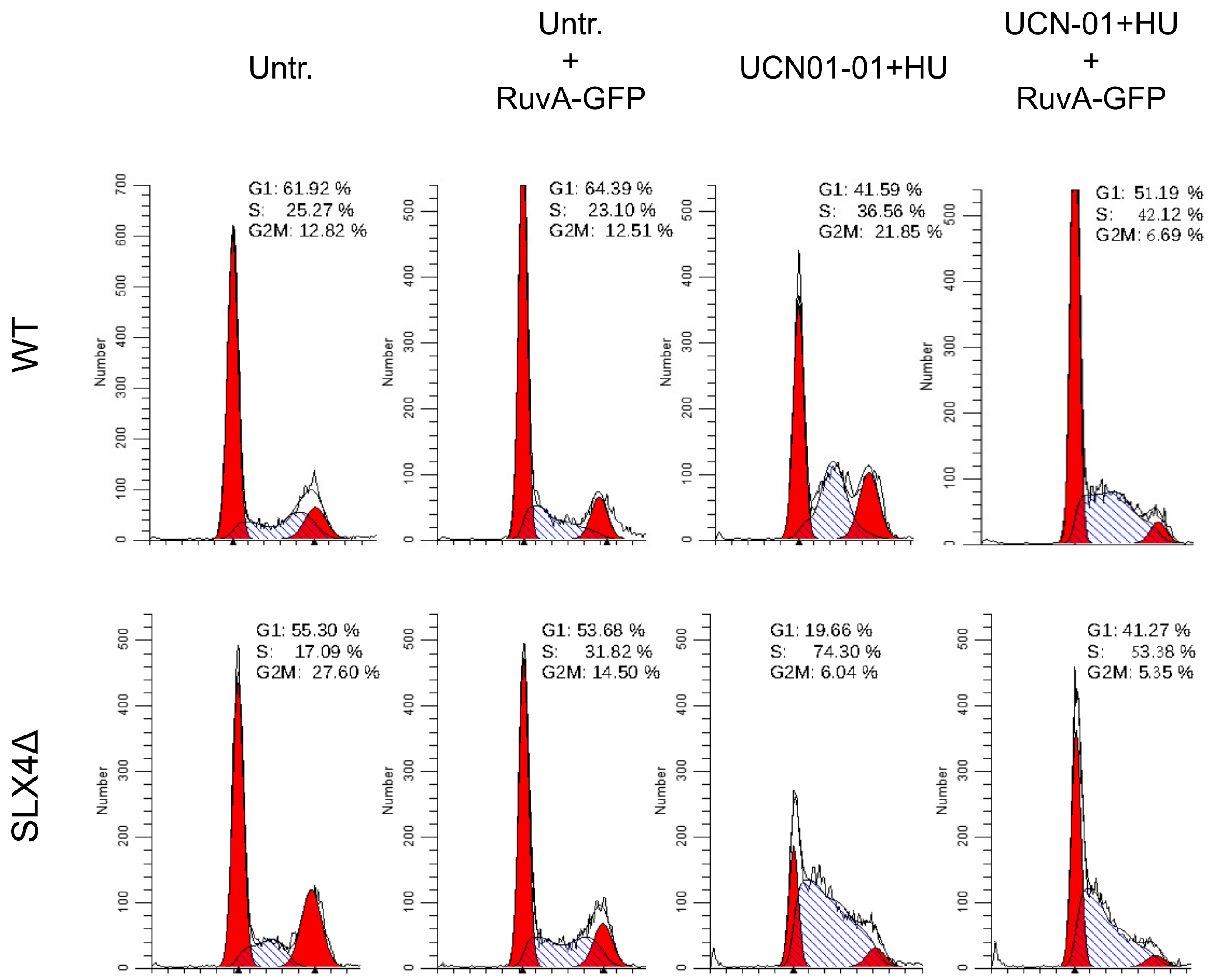

Supplementary Figure 6

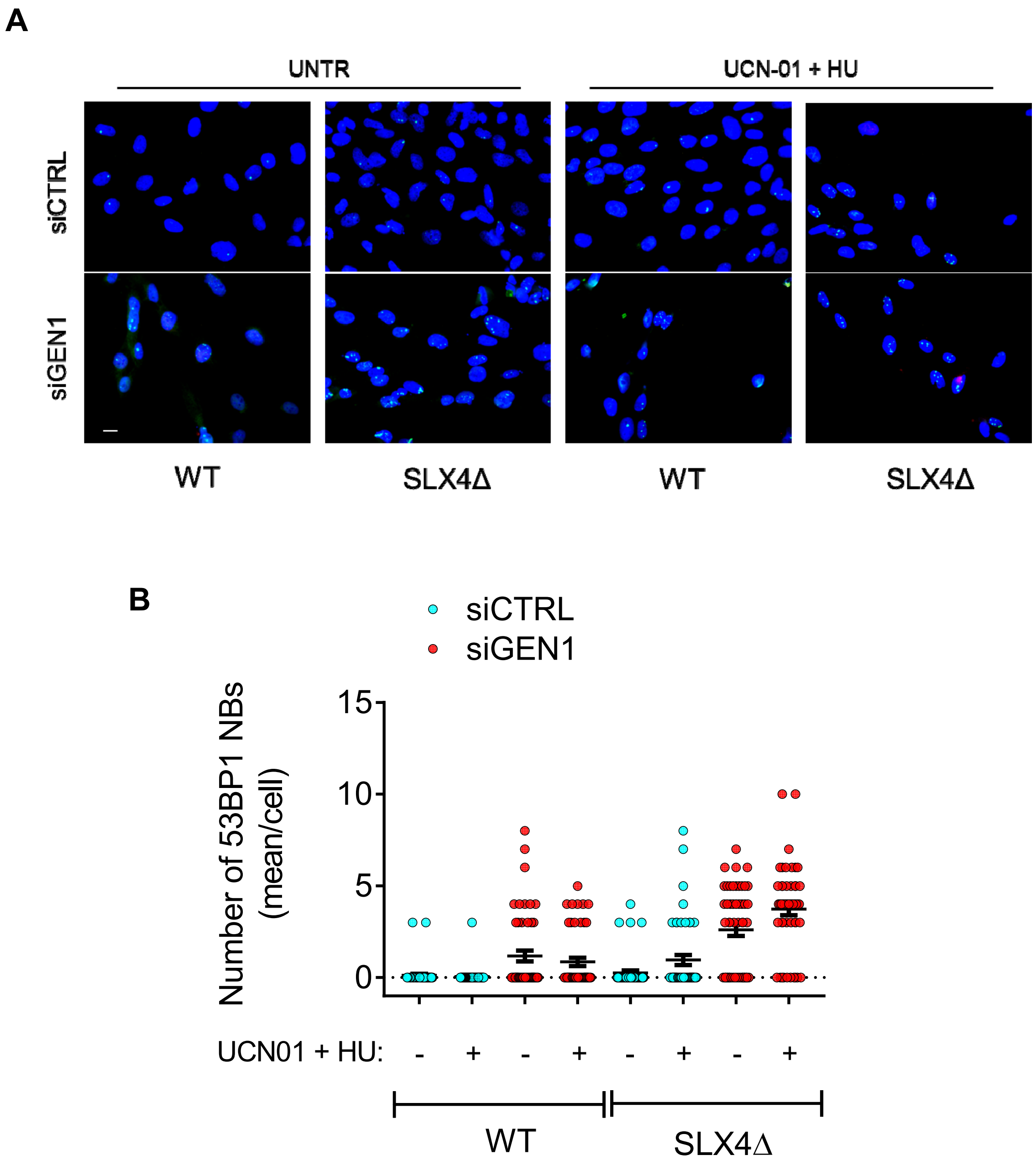

Supplementary Figure 7

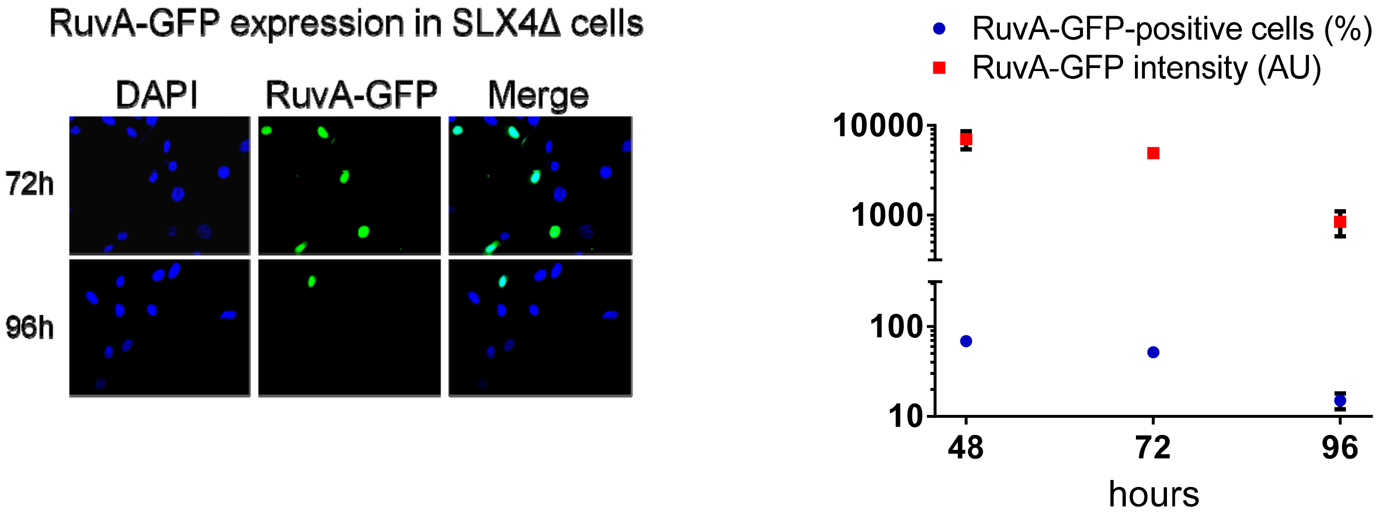

Supplementary Figure 8

Supplementary Figure Legends

Supplementary Figure 1. Depletion of either MUS81 or SLX4 abrogates DSBs formed in response to ICLs. Evaluation of DNA breakage by neutral comet assay. Cells were treated with 1µg/ml MMC for 24h. Data are mean +/- standard errors (SE) from three independent experiments. Representative images from neutral comet assay from selected samples are shown.

Supplementary Figure 2. GEN1 performs DSBs during S-phase. A) GM1604 cells were transfected with siRNAs direct against SLX4 (siSLX4) and 48h after transfection were treated with 600nM UCN-01, 2mM HU, or both for 6 hours. Where indicated, nocodazole was added during the last three hours of treatment to block cells into mitosis. The number of mitotic cells was evaluated by anti-pS10-H3 immunofluorescence. Data are presented as a mean of three independent experiments +/- SE. The top inset shows a Western blotting to evaluate SLX4 depletion. LAMIN B1 was used as a loading control. B) FA-P cells complemented or not with wild-type SLX4 were used to verify formation of DSBs by GEN1 in S-phase. Replication sites were first labeled with CldU for 10min before inducing replication stress and GEN1-dependent DSBs by treating cells with 600nM UCN-01 and 2mM HU for 6hours. To reveal DSBs, an anti-γ-H2AX antibody was used. S-phase cells were detected by anti-CldU antibody (red signal) and overlapping foci (yellow signal) indicate S-phase DSBs. DSBs occurring in S-phase were quantified in each isolated red-positive cell and results expressed as the percentage of CldU/ γ-H2AX colocalising nuclei. Data are presented as means +/- SE of three independent experiments. The images shown in the panel are representative of staining.

Supplementary Figure 3. GEN1 performs DSBs in absence of SLX4. A) FA-P cells complemented or not with wild-type SLX4 were transfected with control siRNA (siCTRL) or siRNA against GEN1 (siGEN1). Western blotting analysis shows the reduction of protein level after 48h from transfection. LAMIN B1 was used as a loading control. B) Cells were treated with 600nM UCN01 and 2 mM HU for 6 h before Comet assay. Graph shows data presented as mean tail moment +/- SE from three independent experiments. Error bars represent standard errors. (ns = not significant $p > 0.05$; **** = $p < 0.0001$, Kruskal-Wallis test).

Supplementary Figure 4. DSBs formed after persistent replication arrest are GEN1-independent in SLX4 depleted cells. A) Western blotting analysis of proteins depletion in GM1604 cells. Cells were transfected with siRNA directed against Ctrl (siCTRL), GEN1 (siGEN1), SLX4 (siSLX4) or MUS81 (siMUS81) alone or in combination with siRNAs against SLX4. LAMIN B1 was used as a loading control. B) Forty-eight hours after transfection, cells were treated with 2mM HU for 24h and DSBs formation was evaluated by Comet assay. Data are presented as mean +/- SE from three independent experiments. (**** = $p < 0.0001$, Kruskal-Wallis test).

Supplementary Figure 5. Expression and chromatin localization of ectopic RuvA-GFP in human cells. A) Representative panel of 293T cells at 48h post-RuvA-GFP transfection. B) Recruitment in chromatin of RuvA-GFP in 293T cells. Cells were transiently-transfected with RuvA-GFP or an empty GFP vector and 48h thereafter were treated with CPT 1µM for 1h to induce DSBs and recombination. After treatment cells were subjected to chromatin fractionation and Western blot analysis shows the amount of RuvA-GFP in the input and chromatin fraction. LAMIN B1 was used as loading control. Fold increase is given over the untreated.

Supplementary Figure 6. RuvA expression rescues S-phase delay in FA-P cells after replication stress. FA-P cells complemented or not with wild-type SLX4 were transfected with RuvA-GFP and 48h thereafter treated with 600nM UCN-01 and 2mM HU for 6h, recovered in fresh medium for an overnight (O/N) and analyzed by flow cytometry after propidium iodide staining for DNA content. Representative flow cytometry profiles are shown. Distribution of cells in each cell cycle phase is reported for each condition.

Supplementary Figure 7. GEN1 depletion increases genome instability in absence of SLX4. A) Immunofluorescence showing accumulation of 53BP1 NBs after recovery from replication fork demise. After 48h from siRNAs transfection, cells were treated with 600nM UCN-01 and 2mM HU for 6h, recovered overnight in drug-free medium and analysed for the presence of 53BP1 NBs. B) The number of 53BP1 NBs per cell was scored in at least 100 nuclei for each condition and reported in the graph as mean +/- SE.

Supplementary Figure 8. Analysis of RuvA-GFP expression with time. A) FA-P cells were transfected with RuvA-GFP and the number of GFP-positive cells assessed by direct fluorescence along with the intensity of fluorescence at different times. Data are presented as means +/- SE of two independent experiments. In same case, SE bars are smaller than the symbol and are not visible in the graph.

Uncropped images of Western blots shown in the main figures

Related to Fig. 1 A

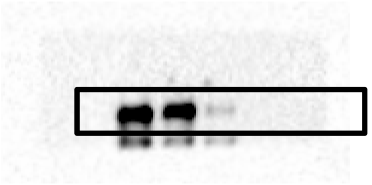

Anti- SLX4

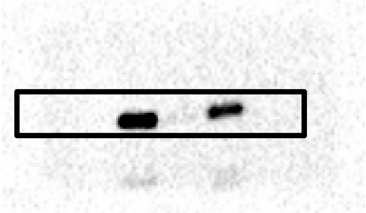

Anti- MUS81

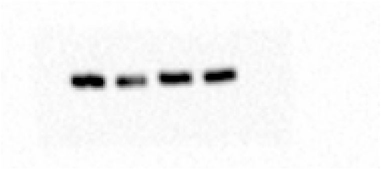

Anti- LAMIN B1

Related to Fig. 1 D

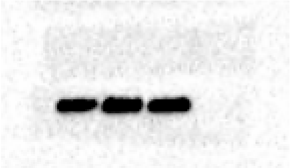

Anti- MUS81

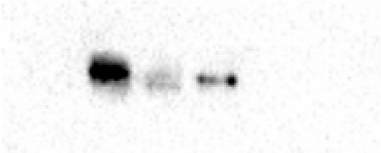

Anti- GEN1

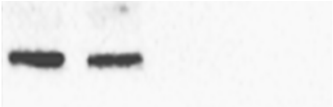

Anti- SLX4

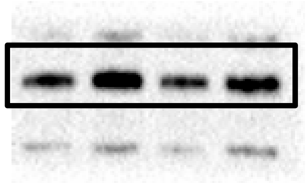

Anti- LAMIN B1

Related to Fig. 2 B

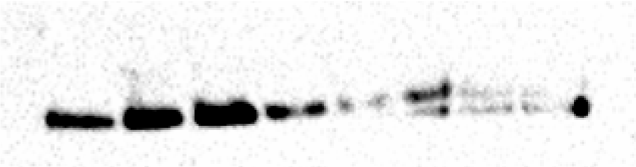

Anti- SLX4

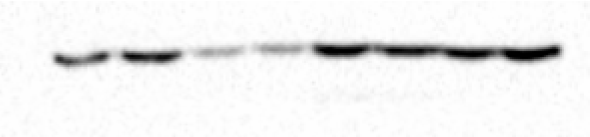

Anti- MUS81

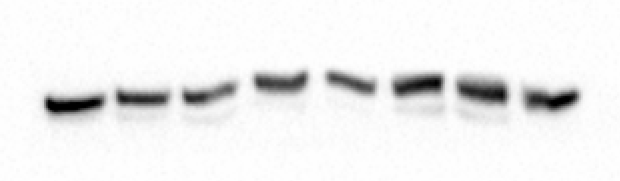

Anti- TOPO II

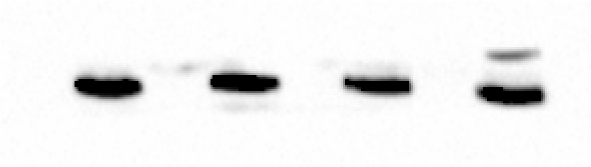

Anti- Cyc E

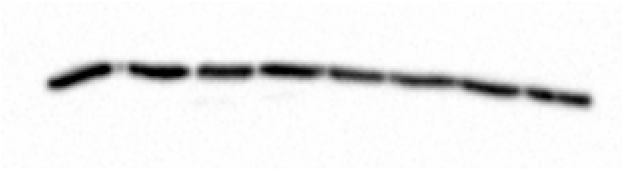

Anti- GAPDH

Related to Fig. 3A

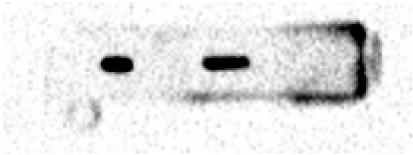

Anti- MUS81

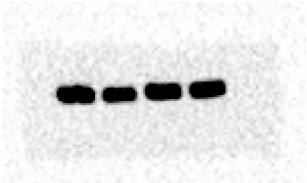

Anti- LAMIN B1

Related to Fig. 3B

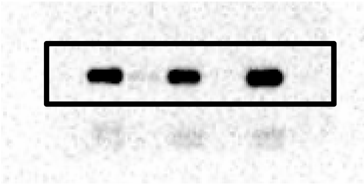

Anti- MUS81

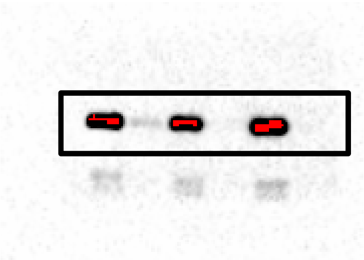

High exposure

Anti- MUS81

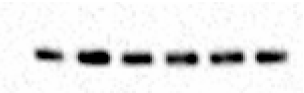

Anti- LAMIN B1

Related to Fig. 4B

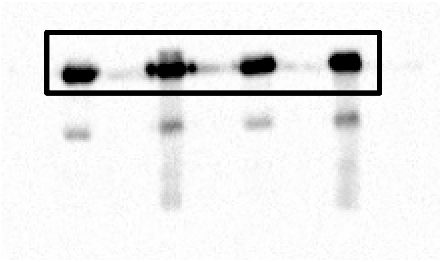

Anti- GFP

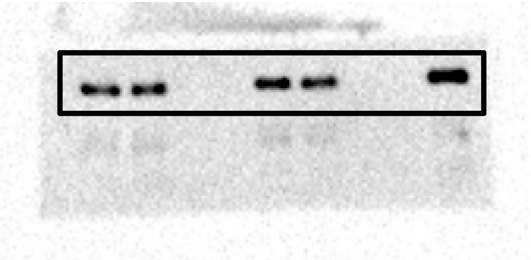

Anti- MUS81

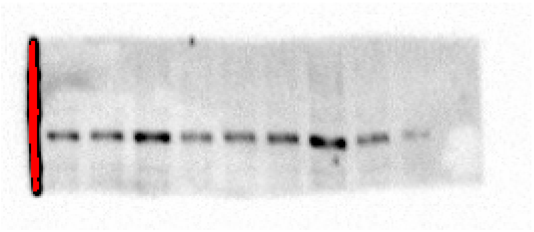

Anti- GEN1

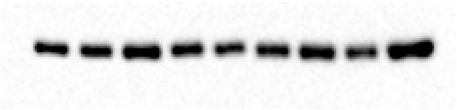

Anti- LAMIN B1

Related to Fig. 4E

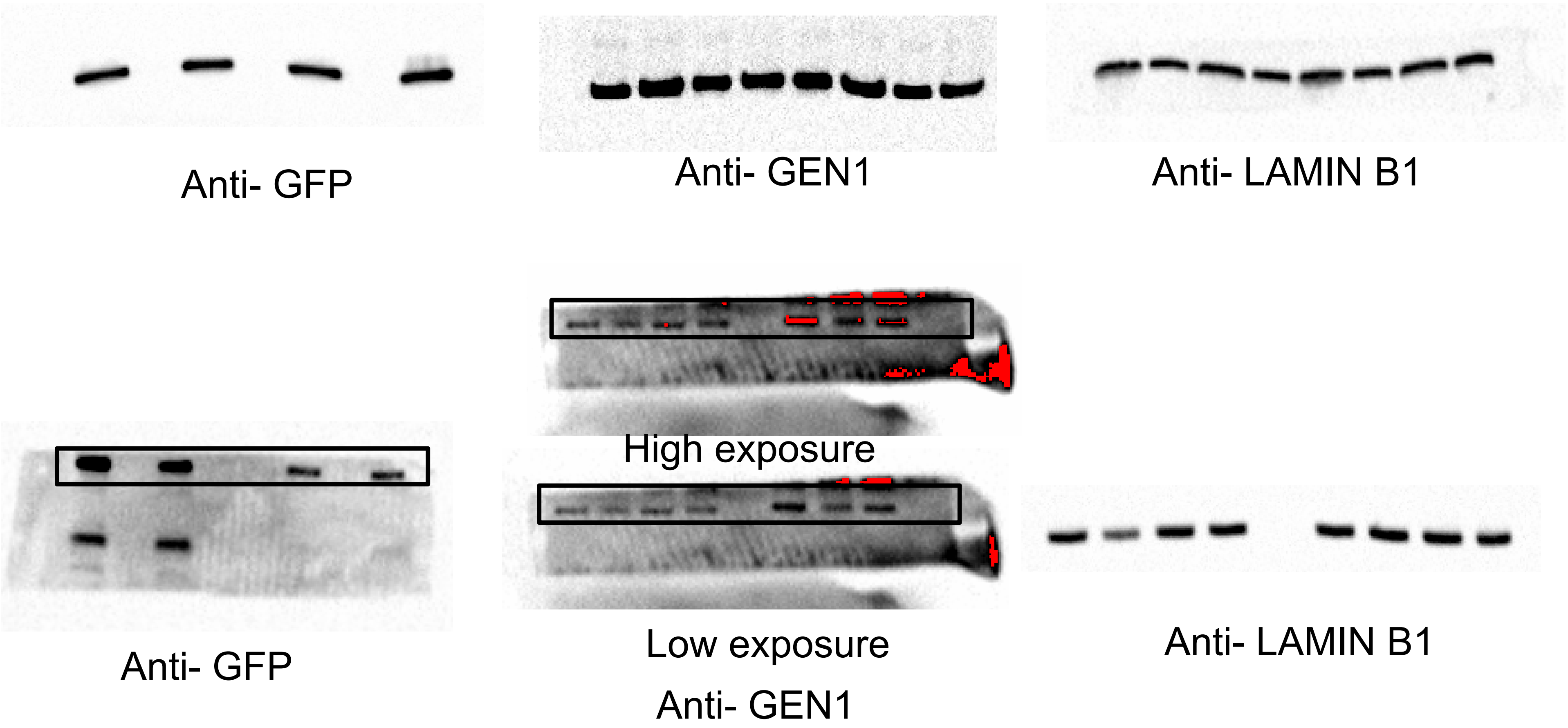

Related to Fig. 5A

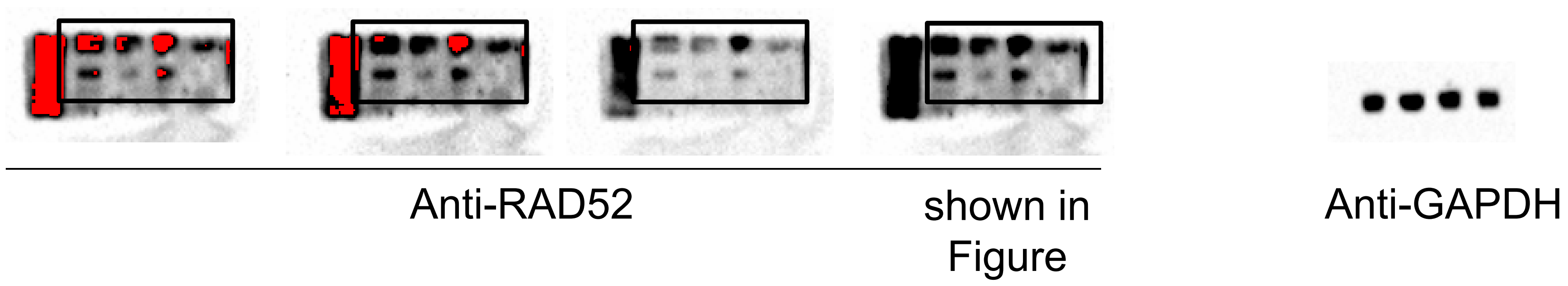

Related to Fig. 5B

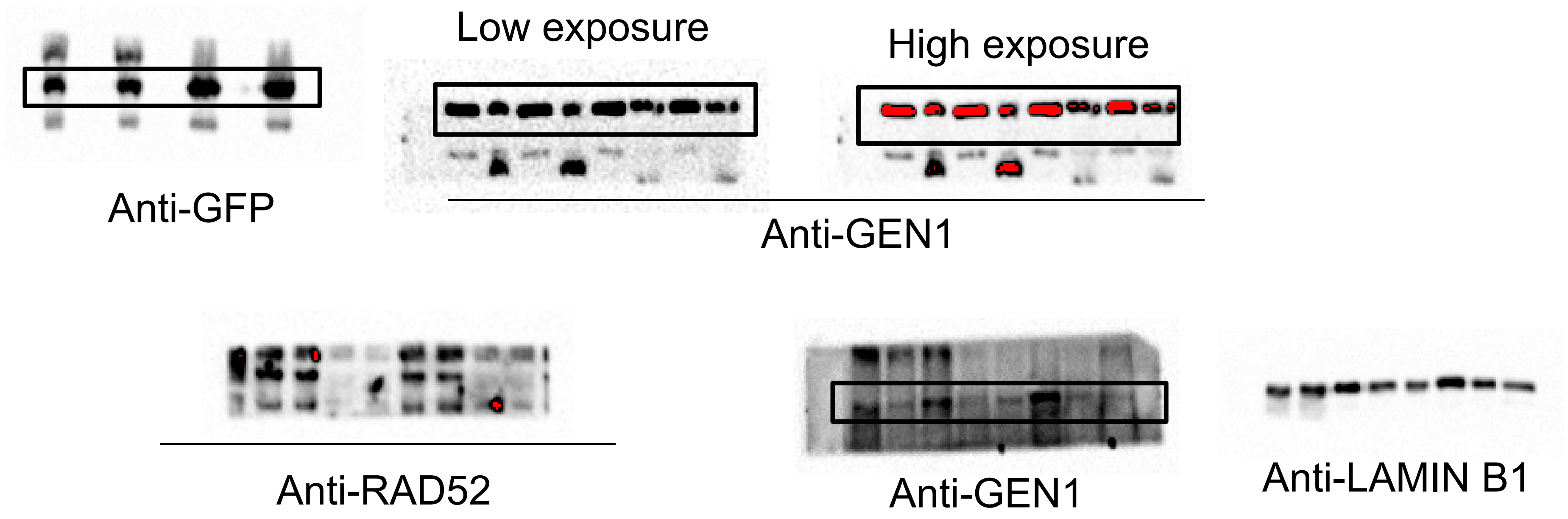

Related to Fig. 5C

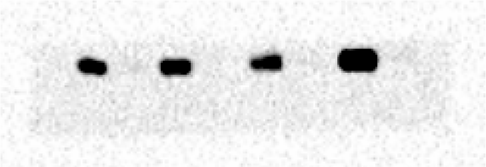

Anti-GFP

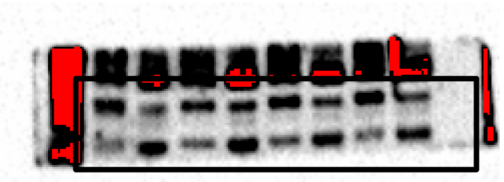

Anti-RAD52

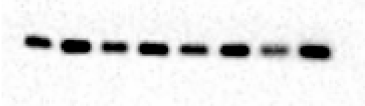

Anti-LAMIN B1

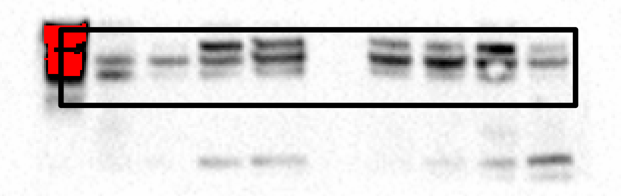

Anti-RAD52

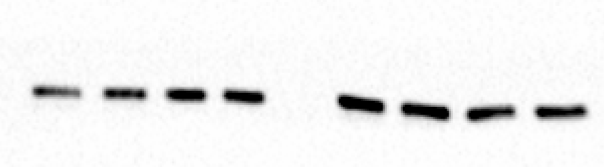

Anti-LAMIN B1

Related to Fig. 6A

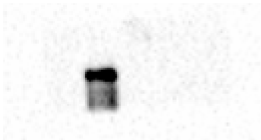
Anti- SLX4

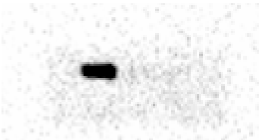
Anti- MUS81

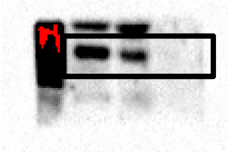
Anti- GEN1

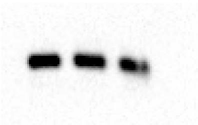
Anti- LAMIN B1

Related to Fig. 6B

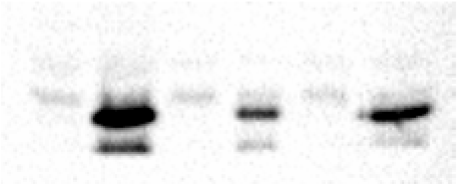
Anti- CycE

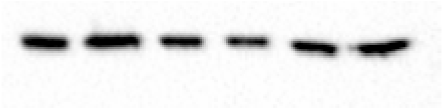
Anti- PCNA

Related to Fig. 6D

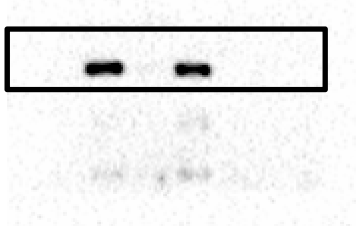

Anti- GEN1

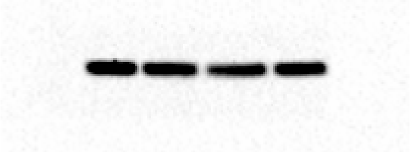

Anti- LAMIN B1

Related to Fig. 7A

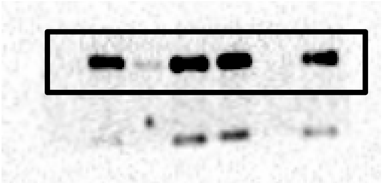

Anti- GEN1

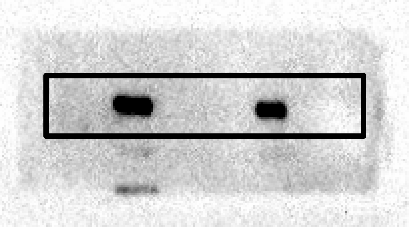

Anti- GFP

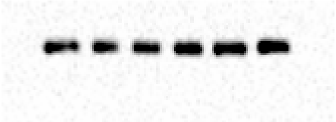

Anti- LAMIN B1

Related to Fig. 8A

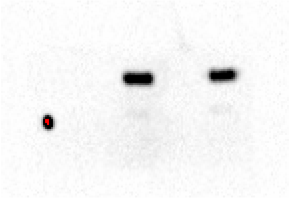

Anti- GFP

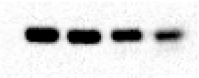

Anti- LAMIN B1
